# Effectiveness of gender-targeted versus gender-neutral interventions aimed at improving dietary intake, physical activity and/or overweight/obesity in young adults (aged 17–35 years): a systematic review and meta-analysis

**DOI:** 10.1186/s12937-020-00594-0

**Published:** 2020-07-30

**Authors:** Thomas Sharkey, Megan C. Whatnall, Melinda J. Hutchesson, Rebecca L. Haslam, Aaron Bezzina, Clare E. Collins, Lee M. Ashton

**Affiliations:** 1 School of Health Sciences, Faculty of Health and Medicine, University of Newcastle, Callaghan, 2308 Australia; 2 Priority Research Centre for Physical Activity and Nutrition, University of Newcastle, Callaghan, 2308 Australia

**Keywords:** Young adults, Gender differences, Nutrition, Physical activity, Obesity, Systematic review

## Abstract

**Background:**

Young adulthood has become synonymous with the development of poor lifestyle behaviours associated with an increased risk of preventable chronic disease in later years. Interventions aiming to improve health behaviours may be more engaging and effective if they are targeted to males or females than interventions with a gender-neutral approach. This review will examine the outcome effectiveness of gender-targeted and gender-neutral interventions targeting nutrition, physical activity or overweight/obesity in young adults (17–35 years).

**Methods:**

Six electronic databases were searched for randomised controlled trials (RCTs) published up to December 2019 that evaluated nutrition, physical activity and/or overweight/obesity interventions in young adults (17–35 years). An effective intervention was one where the change in one or more primary outcome was positive and statistically significantly different from baseline, compared with control, or if no control comparator, compared with another active intervention. Effectiveness of outcomes was compared between gender-targeted and gender-neutral studies.

**Results:**

In total 21,582 manuscripts were identified and 107 RCTs were included; 30 gender-targeted studies (28%) and 77 gender-neutral (72%). Most gender-targeted studies were female targeted (*n* = 22, 73%). Primary outcome/s were adiposity (*n* = 36, 34%), nutrition (*n* = 29, 27%), physical activity (*n* = 28, 26%), or a combination of (*n* = 14, 14%). A greater proportion of gender-targeted than gender-neutral studies were effective in improving nutrition (*n* = 6, 100% and *n* = 17, 74% of studies respectively) and physical activity outcomes (n = 6, 86% and n = 14, 67% respectively), where as a greater proportion of gender-neutral studies were effective in improving adiposity outcomes (*n* = 13, 59% and *n* = 5, 36% respectively). None of these differences were statistically significant. Meta-analyses for weight found no significant differences between gender-targeted and gender-neutral studies for weight loss or weight gain prevention studies. Meta-analysis for fruit and vegetable intake demonstrated a significantly greater increase in intervention participants in gender-targeted studies of +158 g/day for > 3 months.

**Conclusions:**

Although differences in outcome effectiveness were identified between gender-targeted and gender-neutral studies, these were not significantly different. This is likely due to an insufficient number of studies to detect a difference. The meta-analysis for fruit and vegetable intake findings should be interpreted with caution due to including only two gender-targeted studies. The findings collectively are suggestive of a potential difference requiring further investigation. To truly determine the effectiveness of gender-targeted interventions, well-designed RCTs comparing gender-targeted interventions with gender-neutral and control are needed.

**Registration:**

This systematic review is a secondary analysis of studies included in a systematic review examining the effectiveness of interventions targeting nutrition, physical activity, or overweight/obesity in young adults, for which a predefined protocol was registered with PROSPERO (CRD42017075795).

## Introduction

Young adulthood (17–35 years of age) has become synonymous with the development of poor lifestyle behaviours associated with an increased risk of chronic disease in later years [1, 2]. Specifically, rates of obesity are higher in current young adults compared with previous generations. In Australia, overweight and obesity rates have increased from 38 to 46% in the last 6 years in those aged 18–24 years [3, 4]. In the USA the overweight and obesity rate has increased from 42 to 61% from 1994 to 2016 among 20–34-year olds [5]. Contributing to this increased prevalence of overweight and obesity are lifestyle behaviour changes, including worsening dietary patterns and physical inactivity. In a systematic assessment of dietary patterns in adults across 187 countries, 20–29 year olds had the lowest (i.e. least healthy) dietary scores across the two dietary patterns assessed (one reflecting healthy dietary items and one reflecting unhealthy items), compared with all other age ranges [6]. Further, a meta-analysis of physical activity change from adolescence to young adulthood demonstrated a 13–17% decline in physical activity, or 5.2–7.4 min of moderate-vigorous physical activity per day, from age 13 to 30 years [7].

Lifestyle behaviour changes in young adults are influenced by the defining experiences of this life stage, such as changing relationships, living arrangements, study and employment, financial responsibilities, and social environments [8]. These experiences often present barriers for young adults to achieve healthy lifestyle behaviours, such as lack of time and competing interests [8, 9]. However, lifestyle behaviours developed during young adulthood carry on throughout adulthood, and significantly influence an individual’s health trajectory [2, 10, 11]. Therefore, young adulthood is a critical life stage to intervene and establish healthy lifestyle behaviours.

To synthesise the evidence for effective interventions targeting nutrition, physical activity and/or obesity for young adults, several systematic reviews have been conducted [12–15]. For example, in a review of 21 weight gain prevention randomised controlled trials (RCTs) in young adults, significant changes in weight and/or body mass index (BMI) were reported for half of the studies, more commonly those with a theoretical underpinning [14]. Further, in a recent review of eHealth weight management interventions for young adults, eight of 24 studies were found to have positive weight-related outcomes, most commonly web-based and multicomponent intervention approaches [12]. While the reviews to date provide important insight into effective intervention approaches for improving young adults’ health behaviours, it is not yet clear which approaches are most effective. There remain certain intervention aspects which require more focused enquiry, including gender targeting [16].

There are established differences between males and females in terms of their lifestyle behaviours, as well as their motivations and barriers for achieving a healthy lifestyle [8, 9, 17, 18]. In a 2010 systematic review of weight loss interventions in young adults, Poobalan et al. commented on a trend of greater participation by males in exercise training interventions and females in diet and behavioural interventions, noting that further exploration of intervention preferences was warranted [17]. A range of qualitative and quantitative studies have since explored barriers and enablers to healthy lifestyle behaviours in this group [8, 9, 18]. Overall, females generally place greater importance on, and are more motivated towards, healthy eating than males, with key enablers including interest in a healthy diet, and friends and family having, and supporting them to have, a healthy diet [8, 18]. Meanwhile, males generally have more motivators towards being physically active than females, with key enablers including desire for improved body image and fitness [9, 18]. As such, it has been suggested that interventions which are targeted to males or females may be more engaging and effective than interventions with a gender-neutral approach. For example, a 2012 systematic review of male-only weight loss and/or maintenance interventions found that 23 of 31 interventions were effective [19]. Further, a 2015 systematic review of smoking, nutrition, alcohol, physical activity and obesity interventions in young adult males found that six of 10 included studies reported positive, short-term effects for nutrition, alcohol use or multiple risk factors [20]. However, no systematic reviews have evaluated whether gender-targeted interventions or gender-neutral interventions are more efficacious.

This review aims to examine the effectiveness of gender-targeted (including males or females only) versus gender-neutral (including males and females collectively) interventions for improving nutrition, physical activity or overweight/obesity in young adults (aged 17–35 years).

## Methods

### Protocol and registration

This is a secondary analysis of studies included in a systematic review examining the effectiveness of interventions targeting nutrition, physical activity, or overweight/obesity in young adults without gender comparison [21]. The review methods are consistent with the PRISMA (Preferred Reporting Items for Systematic Reviews and Meta-Analyses) guidelines and used a pre-defined protocol registered with PROSPERO (CRD42017075795). Results pertaining to the nutrition and obesity outcomes have been published [21, 22].

### Eligibility criteria

#### Types of participants

Participants were aged 17–35 years to align with the international definition of young adulthood [23]. Studies with participants from groups with diagnosed conditions linked to obesity risk factors (e.g. type 2 diabetes) or from special populations (e.g. severe mental illness, eating disorders, elite athletes, and pregnant women) were excluded as the objective of this review is to provide recommendations to the broader healthy young adult population.

#### Types of interventions

Included interventions were those with the primary objective of improving nutrition or physical activity, or treating or preventing obesity and designed to promote behaviour change. Studies which primarily aimed to improve the acute outcomes of weight loss on other clinical biomarkers (e.g. insulin) were excluded along with studies exploring the impact of weight loss surgery or anti-obesity medications. Additionally studies utilising supervised and controlled exercise programs examining the impact of exercise on clinical biomarkers and fitness and not designed to promote exercise behaviour change were excluded.

#### Types of comparators

Any comparator or control were considered for inclusion, including comparisons with no-intervention (e.g. waitlist control) and/or compared to active treatment arms.

#### Type of outcome measures

The type of outcomes in included studies were nutrition (e.g. serves of fruit and vegetables, serves of discretionary foods, energy consumption), physical activity (PA) (e.g. minutes walking, sedentary time, light, moderate and vigorous intensity physical activity) and adiposity outcomes (e.g. body mass index, weight, percent body fat). Outcomes could be measured by any methods. A minimum of baseline and one post-test measurement were required for inclusion.

#### Types of studies

Included studies were RCTs, including feasibility and pilot RCTs, published in the English language.

### Information sources and search

Electronic searches were conducted in six online databases; MEDLINE (Ovid), EMBASE (Ovid), PsycINFO (Ovid), Science Citation Index (WoS), Cinahl (EbscoHost) and Cochrane Library (Wiley) from the date of inception to December 2019. Search terms with appropriate truncation and indexing were used to identify articles eligible for inclusion. The search strategy aimed to identify studies in young adults to improve their diet, physical activity or prevent or treat obesity, hence search terms including; ‘young adult’, ‘college aged’, ‘university student’, ‘diet’, ‘healthy eating’, ‘exercise’, ‘physical activity’, ‘weight loss’ and ‘obesity’ were used. The search strategy was revised for each individual database. The full detailed search strategy is included as Table S1 (Additional File). The electronic search was augmented by the addition of hand searches of journals and reference lists of relevant articles.

### Study selection

Title, abstract and keywords of all identified papers were assessed by two independent reviewers (LMA and MJH or MCW or CEC or TS or RLH). The full text of potentially relevant titles and abstracts were screened by two independent reviewers (LMA and MCW or TS or RLH), with a third reviewer used to resolve disagreements (MJH). Where papers did not provide sufficient details to determine eligibility (e.g. age range), primary authors were contacted via email to determine if inclusion criteria was met. If the author did not respond within 1 month, the study was subsequently excluded. Reasons for exclusion were recorded for ineligible papers.

### Risk of bias

In order to determine the risk of bias in the studies design, the methodology was graded according to the Cochrane Collaboration’s Tool for assessing risk of bias [24]. The tool measures indicators of internal validity including: sequence generation, allocation concealment, blinding of participants, incomplete outcome data, selective outcome reporting and other undefined risks of bias. The bias check was conducted by two independent reviewers (LMA and MJH or AB or RLH or MCW). A third reviewer was consulted in any cases of disagreement (MJH or TS).

### Data extraction

Data was extracted by one reviewer (TS or RC) and checked by a second reviewer (LMA or MJH or AB or RLH or MCW). Data was extracted relating to study characteristics (e.g., authors, title, and date of publication, country, and duration), study methodology (e.g. intervention method, intervention duration, study arms), sample characteristics (e.g. number, sex, age, ethnicity), recruitment and retention (e.g. rates, setting) and study results (e.g. physical activity, nutrition, adiposity outcomes). Study results were reported for the primary outcome/s only. The studies primary outcome, if it was not clearly stated as either an outcome or aim, was determined by considering the basis of the sample size calculation, or if it remained unclear, equal weighting was given to all included outcome measures.

### Synthesis of results and analytic strategy

#### Narrative summary

Results are presented narratively for all studies. Studies were considered gender-targeted if participants were exclusively male or female. The gender-neutral interventions were those where participants were both male and female. An effective intervention was defined as one where the change in one or more primary outcome was positive and statistically significantly different from baseline, compared with control, or if no control comparator, compared with another active intervention. Studies are described according to whether the primary outcome/s were nutrition, physical activity or adiposity outcomes, or a combination of these outcomes. Differences between gender-targeted and gender-neutral studies were assessed for study characteristics, risk of bias components, and effectiveness using Chi-square tests/Fishers exact test for categorical variables, depending on frequency per category, and t-tests/Wilcoxon Mann-Whitney tests for continuous variables, depending on the normality of the data.

#### Meta-analysis

Meta-analysis was performed for outcomes where there were sufficient comparable studies, i.e. at least two gender-neutral and gender-targeted studies which reported values as mean and SD at all time points. Meta-analysis was performed using R statistical software (V 3.5.1, Vienna, Austria) using the Metafor package (V 2.0, Vienna, Austria). Meta-analyses were performed to assess change in fruit and vegetable intake (g/day) and weight (kg) for both intervention and comparator/control groups at each time point. Fruit and vegetable intakes were first standardised to an intake in grams per day. For studies where portions per day or serves per day were reported, a standardisation procedure was used whereby the exposure level was multiplied by 106 g to give grams per day, to be consistent with previous meta-analyses [25–27]. Six studies from the USA reported fruit and vegetables in cups per day, and 160 g was used as a cup equivalent size as the USA serving of one-half cup of vegetables or a medium-size piece of fruit was taken to equal 80 g [26]. For studies reporting fruit and vegetable intakes separately, the results were converted by summing the mean intakes for fruit and for vegetable to give a total mean. For the SD and sample size, a conservative approach was taken assuming the Standard Error (SE) for each mean were different. The SD combined score was determined as [n1.SE1^2^ + n2.SE2^2^]^½^ and sample size was as reported for the study. Where SDs were reported rather than SEs, the SDs were used in place of the SEs and n1, n2 was set at 1. The studies with weight as a primary outcome were split into weight gain prevention and weight loss studies for analyses.

For each study, the effect at baseline and each time point, expressed as months post baseline, was estimated as the mean difference (mean intervention group − mean control group) using the unbiased option for variance estimates. Uncertainty in the mean estimates was expressed as 95% confidence intervals. To account for multiple measures per study a series of multilevel models were investigated with nested random effects top level being study, then treatment type and time period using REML estimation. The final model contained study and time as nested random effects due to the treatment type random effect having zero variance. Moderator variables evaluated as fixed effects in the model included treatment type (simplified as gender-neutral or gender-targeted) and time treated as a categorical variable in two versions (as originally reported with 12 time-points for fruit and vegetables, 8 time points for weight outcome in weight gain prevention studies and 11 time points for weight outcome in weight loss studies) and a simplified grouped form with baseline, up to 3 months and greater than 3 months, with interaction between these two tested. There was substantial correlation between time-periods, with the time random effect similar in size to study random effects. Hence, the moderator effect for the three-group version of time was estimated at each of the two follow-up time periods as difference from baseline. To examine homogeneity of variance and normality assumptions, residual plots were used. To assess presence of publication bias, funnel plots were created and visually inspected, and to test for funnel plot asymmetry, rank correlation was used. Heterogeneity between studies was observed from the forest plots and differences between averages over time was observed from CI plots.

## Results

### Description of included studies

**Fig. 1:**
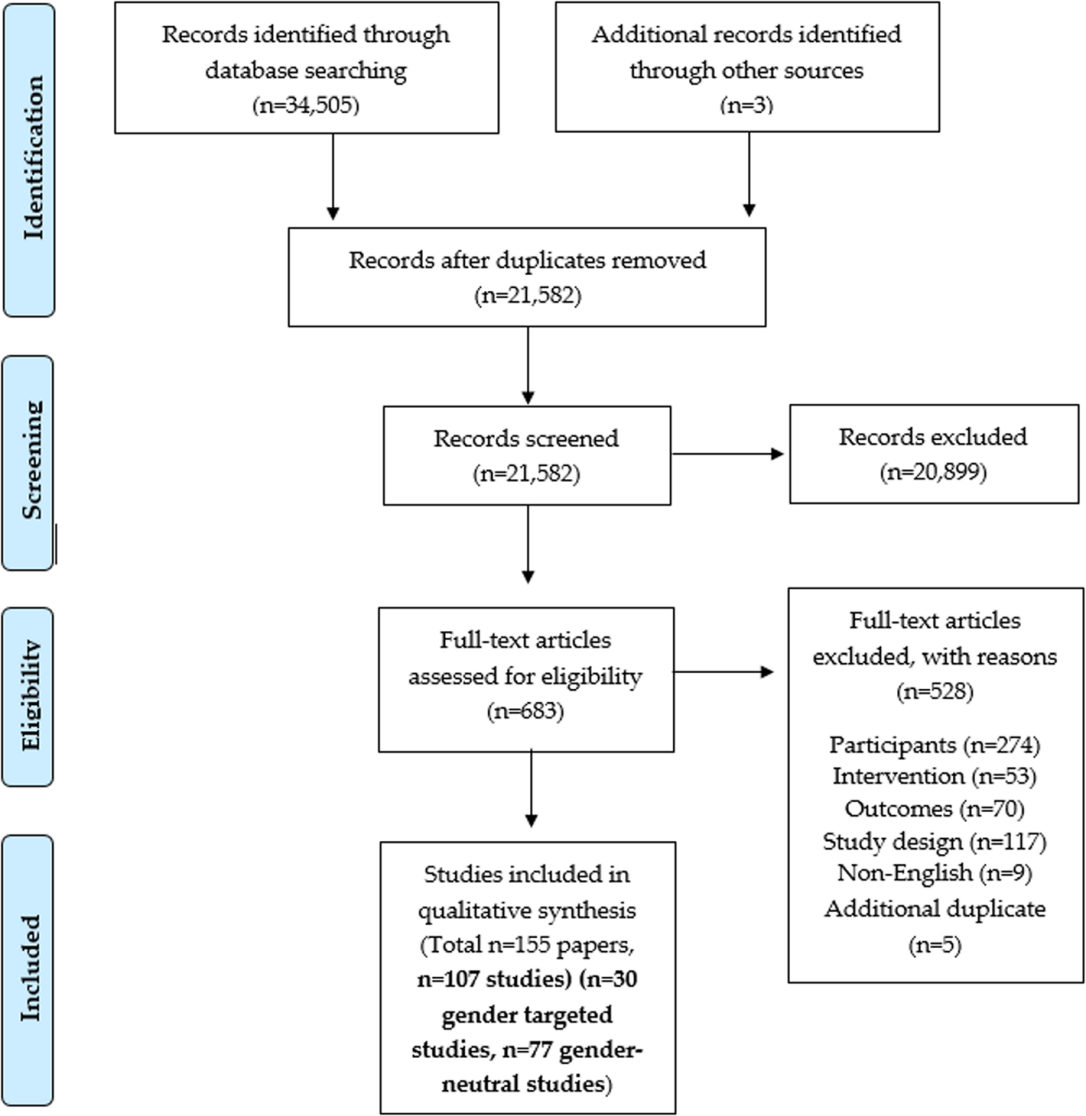
PRISMA flow diagram of included studies

Following screening of 21, 582 manuscripts identified, 107 individual RCTs were included from a total of 155 individual papers (Fig. 1). Thirty studies were gender-targeted (28%) and 77 were gender-neutral (72%). Study characteristics are summarised in Table 1, with detailed characteristics presented in Supplementary Table 2. The majority of included studies have been published since 2013 (*n* = 73, 68%), and this was consistent for both gender-targeted and gender-neutral studies (*n* = 18, 60% and *n* = 55, 71% respectively). Fifty-four percent of included studies were conducted in the USA (*n* = 58, 54%), which was the most common country for both the gender-targeted (*n* = 15, 50%) and gender-neutral studies (*n* = 43, 56%). Universities were the most common recruitment setting among both gender-targeted and gender-neutral studies (*n* = 19, 63% and *n* = 65, 84% respectively). The average retention rate at the point of longest follow up was 74%, and this was consistent for gender-targeted and gender-neutral studies (69 and 76% respectively).

**Table 1:** Summary of study characteristics in 107 studies of nutrition, physical activity and obesity interventions in young adults, by gender-targeted versus gender-neutral studies

	Total (***n*** = 107)		Gender-targeted (***n*** = 30)		Gender-neutral (***n*** = 77)	
	n	%	n	%	n	%
Publication year n (%) ^a^						
Before 1999	2	2	2	7	0	0
1999–2002	4	4	3	10	1	1
2003–2007	6	6	3	10	3	4
2008–2012	22	21	4	13	18	23
2013–December 2019	73	68	18	60	55	71
Country n (%)						
United States	58	54	15	50	43	56
Australia	11	10	3	10	8	10
Canada	8	7	2	7	6	8
UK	5	5	0	0	5	7
Thailand	3	3	1	4	2	3
Finland	3	3	3	11	0	0
New Zealand	2	2	0	0	2	3
Italy	2	2	0	0	2	3
Other	14	13	5	17	9	12
Number of participants						
Total	29,566		10,400	35	19,166	65
Mean	276.3		346.7		248.9	
Median	124		81		150	
Range	20–3336		32–3336		20–2024	
Sex n (%) ^a^						
Female	17,294	60	5095	49	12,199	65
Male	11,750	40	5302	51	6448	35
Age						
Mean years (SD)	21.1	2.9	21.1	3.6	21.1	2.7
17- ≤25 years	59	55	15	50	44	57
17- ≤30 years	23	21	7	23	16	21
17- ≤35 years	24	22	8	27	16	21
Not reported	1	1	0	0	1	1
Ethnicity n (%)						
Predominantly white	62	58	13	43	49	64
Predominantly non-white	7	7	1	3	6	8
Not reported	38	36	16	53	22	29
Recruitment setting n (%) ^a^						
College/University	84	79	19	63	65	84
Community	10	9	2	7	8	10
Health service	2	2	2	7	0	0
Workplace	1	1	1	3	0	0
Military	4	4	4	13	0	0
College/University with other setting	5	5	2	7	3	4
Not reported	1	1	0	0	1	1
Focus of primary outcome/s n (%)						
Nutrition	29	27	6	20	23	30
Physical Activity	28	26	7	23	21	27
Obesity	36	34	14	47	22	29
Nutrition & Physical Activity	6	6	1	3	5	6
Obesity, Nutrition & Physical Activity	5	5	1	3	4	5
Obesity & Physical Activity	3	3	1	3	2	3
Intervention focus n (%) ^a^						
Nutrition	48	28	7	17	41	31
Physical Activity	50	29	13	31	37	28
Obesity	11	6	7	17	4	3
Nutrition & Physical Activity	10	6	1	2	9	7
Obesity, Nutrition & Physical Activity	31	18	7	17	24	18
Obesity, Nutrition &/or Physical Activity + other	24	14	7	17	17	13
Mode of intervention delivery n (%)						
Face-to-face only	38	22	12	29	26	20
eHealth only	61	35	8	19	53	40
Print materials only	4	2	0	0	4	3
Wearable device only	2	1	1	2	1	1
Face-to-face + print materials	22	13	9	21	13	10
Face-to-face + eHealth	21	12	6	14	15	11
Face-to-face + eHealth + wearable device	2	1	0	0	2	2
Face-to-face + eHealth + print materials	8	5	2	5	6	5
Face-to-face + wearable device	2	1	1	2	1	1
eHealth + wearable device	7	4	3	7	4	3
eHealth + print materials	4	2	0	0	4	3
Wearable device + print materials	2	1	0	0	2	2
Study arms ^a^						
Total	261		70	27	191	73
Active	173		42	24	131	76
Intervention duration (weeks) ^a^						
Mean	15.5		15		15.7	
Median	8.0		10.0		8.0	
Range	< 1–156		< 1–130		< 1–156	
Retention rate						
Post-intervention (%)	83%		82%		83%	
Range	23–100%		33–100%		23–100%	
At longest follow-up point (%)	74%		69%		76%	
Range	8–100%		8–100%		11–98%	

^a^Statistically significant difference between gender-targeted and gender-neutral studies (*p* < 0.05)

#### Description of participant demographic characteristics

A total of 29,566 participants were included across the 107 studies. Study participant numbers in individual studies ranged from 20 to 3336, with a mean participant number of 276. This was higher in gender-targeted than gender-neutral studies with 347 and 249 participants respectively, however was not significantly different. In the gender-neutral studies, 65% of participants were female. In the gender-targeted studies 22 studies (73%) were female targeted compared with eight male targeted studies (27%). The most common age range of participants was 17–25 years, in 59 (55%) studies. This was not significantly different among gender-targeted (*n* = 15, 50%) and gender-neutral studies (*n* = 44, 57%). Participant ethnicities were reported in 69 studies (65%), and were predominantly white/Caucasian overall (*n* = 62, 58%), and in the gender-targeted (*n* = 13, 43%) and gender-neutral studies (*n* = 49, 64%).

#### Description of interventions

Two hundred and sixty-one study arms were reported across the included studies. Of these, 173 were active intervention arms, with the remaining being no intervention/waitlist or alternative intervention control groups. Across all studies, the focus of the active study arms was most commonly physical activity (*n* = 50, 29%), followed by nutrition (*n* = 48, 28%), and obesity, nutrition and physical activity (*n* = 31, 18%). This was significantly different between the gender-targeted and gender-neutral studies (*p* = 0.014), with the most common focus among gender-targeted studies being physical activity (*n* = 13, 31%), and the most common among gender-neutral studies being nutrition (*n* = 41, 31%). The most common mode of delivery utilised was eHealth (including email, mobile phone applications, websites and social media) used in 35% (*n* = 61) of the active arms, followed by face-to-face used in 22% (*n* = 38). In the gender-targeted interventions, face-to-face was the most common mode of intervention delivery used in 29% of interventions (*n* = 12), while eHealth was the most common mode of delivery in the gender-neutral interventions (*n* = 53, 40%), but this was not significantly different. The mean intervention duration was 15.5 weeks; which was significantly higher in gender-neutral than gender-targeted studies; 15.7 and 15.0 weeks respectively (*p* = 0.02).

#### Description of study outcomes

Of the 107 included studies, results were recorded for 190 separate primary outcome measures, with 39 studies having more than one primary outcome. Most commonly, primary outcome/s were adiposity outcomes (*n* = 36, 34%), while 29 studies had nutrition related primary outcome/s (27%) and 28 for physical activity (26%). The remaining studies had more than one primary outcome, including nutrition and physical activity (*n* = 6, 6%), adiposity, nutrition and physical activity (*n* = 5, 5%), or adiposity and physical activity outcomes (n = 3, 3%). Gender-targeted studies mirrored the overall studies with adiposity outcomes being the most commonly reported primary outcome (*n* = 14, 47%). However, nutrition was the most commonly reported primary outcome among the gender-neutral studies (*n* = 23, 30%), but this was not significantly different.

### Risk of Bias

**Fig. 2:**
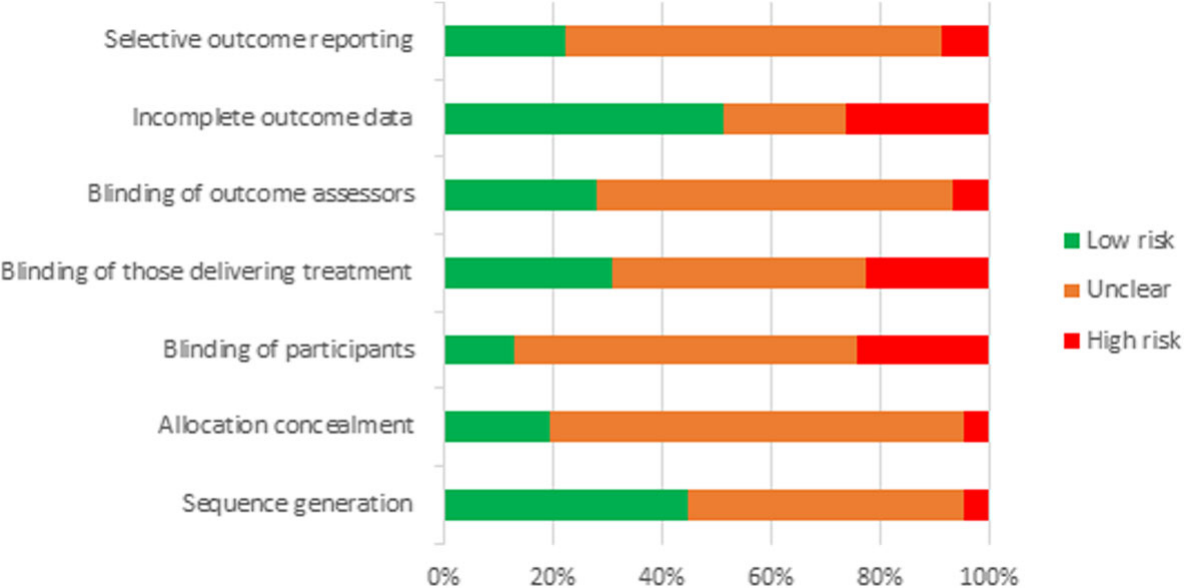
Percentage of studies from risk of bias assessment categorised as low, unclear or high risk, by individual risk components

The risk of bias assessment is summarised in Fig. 2. There were no significant differences for any of the individual risk of bias components between gender-targeted and gender-neutral studies. As such, results are presented for all studies together. Half of the studies provided insufficient detail regarding generation of the allocation sequence (*n* = 54, 50% unclear), and most failed to describe allocation concealment methods (*n* = 81, 76% unclear). There was a high or unclear risk of bias for most studies for blinding of participants (*n* = 93, 87%), blinding of those delivering the intervention (*n* = 74, 69%) and blinding of outcome assessors (*n* = 77, 72%). Half of the studies (*n* = 52, 48%) failed to adequately described study attrition and any exclusions from the analysis. Most studies also provided insufficient detail to determine if they were free of selective outcome reporting (n = 74, 69% unclear).

### Nutrition outcomes

#### Outcome effectiveness: narrative summary

Nutrition and dietary behaviours were the primary outcome in 29 included studies (27%). Of these, 23 studies (79%) reported a significant between group difference and were classified as effective (Table 2). Most commonly, studies targeted fruit and/or vegetable intake (*n* = 22, 76%), and of these, 17 studies were effective (77%).

**Table 2:** Outcome effectiveness in 107 studies of nutrition, physical activity and obesity interventions in young adults

		Outcome effective n (%)					
		Total		Gender-targeted		Gender-Neutral	
	Specific outcome	n	%	n	%	n	%
Nutrition (*n* = 29)	Fruit and/or vegetable intake (*n* = 22)	17	77	2	100	15	75
	Energy intake from fat (*n* = 2)	1	50	1	100	0	0
	Micronutrient intake (*n* = 2)	2	100	1	100	1	100
	Whole grain bread intake (*n* = 1)	1	100	1	100	0	0
	Diet behaviour score (*n* = 2)	1	50	1	100	0	0
	Meat consumption (*n* = 2)	2	100	1	100	1	100
	**Total effective studies**	**23**	**79**	**6**	**100**	**17**	**74**
Physical activity (*n* = 28)	Time in physical activity (*n* = 17)	10	59	3	75	7	54
	Steps per day (*n* = 6)	5	83	3	100	2	67
	Gym attendance (*n* = 2)	2	100	1	100	1	100
	Met PA guidelines (*n* = 1)	1	100	0	0	1	100
	Exercise frequency (*n* = 4)	3	75	0	0	3	75
	PA score (time x frequency) (*n* = 1)	1	100	0	0	1	100
	**Total effective studies**	**20**	**71**	**6**	**86**	**14**	**67**
Obesity (*n* = 36)	Weight (*n* = 29)	16	55	5	42	10	59
	BMI (*n* = 11)	4	36	1	33	4	50
	Waist Circumference (*n* = 4)	1	25	0	0	1	50
	Visceral fat area (*n* = 1)	1	100	0	0	1	100
	**Total effective studies**	**18**	**50**	**5**	**36**	**13**	**59**
Nutrition and Physical activity (*n* = 6)	Met PA guidelines (*n* = 1)	1	100	0	0	1	100
	Exercise frequency (*n* = 2)	2	100	0	0	2	100
	Time in PA (*n* = 2)	1	50	0	0	1	50
	Fruit and/or vegetable intake (*n* = 4)	2	50	0	0	2	50
	Energy intake from fat (*n* = 2)	2	100	0	0	2	100
	**Total effective studies**	**5**	**83**	**0**	**0**	**5**	**100**
Obesity, Nutrition and Physical activity (*n* = 5)	Fruit and/or vegetable intake (*n* = 3)	3	100	0	0	3	100
	Time in PA (*n* = 3)	1	33	0	0	1	50
	**Total effective studies**	**3**	**60**	**0**	**0**	**3**	**75**
Obesity and Physical activity (*n* = 3)	Time in PA (*n* = 2)	1	50	0	0	1	50
	**Total effective studies**	**1**	**33**	**0**	**0**	**1**	**50**
**All studies (*n*** **= 107)**	**Total effective studies**	**70**	**65**	**17**	**57**	**53**	**69**

No statistically significant differences between gender-targeted and gender-neutral studies (*p* > 0.05)

Among the gender-targeted studies, all six (100%) were effective. Franko et al. found that females who completed a two week online nutrition education program reported greater increases in fruit and vegetable intake compared with an attention control group at 3-month follow-up (+ 0.45 serves/day, *p* = 0.002) [28]. Compared with no intervention control groups, Williams et al. demonstrated a greater reduction in percentage energy intake from fat at 6-week follow-up in males who received 4 weeks of nutrition counselling (− 3.4%, *p* = 0.02) [29], and Tavakoli et al. reported a greater increase in dietary behaviour score at 4-week follow-up among males who received a two session theory based nutrition education (+ 1.07, *p* < 0.05) [30]. Providing nutrition education and increasing the availability of vegetables and wholegrain bread in the military setting was also effective compared with a no intervention control, with males reporting greater increases in vegetable (+ 137 g/day, *p* < 0.001) and whole grain bread intakes (+ 56 g/day, *p* < 0.001) post the 5-month intervention [31]. Amiot et al. reported a greater decrease in meat consumption among males who received an education and goal setting intervention compared with a no intervention control group (− 64 g/day, *p* < 0.05) at 4-weeks following the 2-week intervention [32]. Jung et al. reported a greater increase in calcium intake in females who received positively framed, targeted education materials compared with a general education intervention at 12-months following a 14 week intervention (+ 295 mg/day, *p* < 0.01) [33].

Of the 23 gender-neutral studies, 17 were effective (74%). Fifteen studies reported a significant difference in fruit and/or vegetable intake (75%) [34–47], one reported a significant difference in micronutrient intake [48], and one in meat consumption [49] favouring the intervention. Two studies compared interventions against attention control groups (e.g. anatomy education website), including providing fruit and vegetables with/without text message support, and an online nutrition and physical activity education program with/without a booster session [34, 41]. These studies reported 0.24–0.91 serves/day greater increases in fruit and vegetable intake than controls post the 2-week interventions [34, 41]. Three studies compared interventions where participants received nutrition education plus personalised feedback, phone call support or a psychological component, with general nutrition education only [39, 40, 44]. Intervention participants reported greater odds of meeting vegetable intake guidelines immediately following a 4-week intervention (OR = 2.93, *p* = 0.04) [39] and greater increases in fruit and/or vegetable serves/day at 12-months following a 24 week intervention (0.15 serves/day) [40] and at 6-weeks following a single session intervention (0.49 serves/day) [44]. In three studies, single session interventions where participants planned their fruit and/or vegetable intake resulted in greater increases in serves/day at 1-week follow-up compared with no intervention controls (0.3–1.03 serves/day) [36, 42]. While another single session intervention study found that planning and mentally rehearsing fruit intake in relation to a goal was more effective than setting a goal only at 1-week follow-up (+ 0.23–1.19 fruit portions/day) [43]. Nix et al. reported a + 0.5cups/day greater increase in fruit and vegetable intake at 1-week follow-up in participants who received a single message indicating that their peers consume more fruit and vegetables than them, as opposed to a message indicating their peers consume less [47]. Richards et al. reported greater serves/day in participants who received tailored nutrition education content and a motivational interview compared with no intervention controls (+ 0.6 serves/day) at the end of the 16-week intervention [45]. Brown et al. and Rompotis et al. reported greater increases in fruit intake at the end of the 7 and 8-week interventions where participants received nutrition education via text messages compared with print materials, and where messages focused on habit formation rather than general healthy eating, however actual intakes were not reported [35, 46]. Lhakhang et al. reported a 2.08 serves/day greater intake of fruit and vegetables at 5-week follow up in participants who received a motivational intervention first followed by a self-regulatory intervention over 2.5-weeks, as opposed to the reverse sequence [37]. Meng et al. found that participants who self-tracked their fruit and vegetable intake via eHealth in a group environment, compared with individually, reported greater increases in fruit and vegetable intake (+ 1.69–2.02 serves/day) at the end of the 4-week intervention [38]. Goodman et al. found that, compared with no intervention, participants who completed a 12-week online intervention involving education and self-tracking reported greater increases in Vitamin D intake immediately post intervention (+ 177 IU, *p* < 0.001) [48]. Carfora et al. reported a 1 serve/week lower intake of red and processed meat at 10-week follow up in participants who received a 2-week intervention of daily messages focusing on the negative impacts of meat consumption compared with a purely information message, and a control message focusing on sugar consumption [49].

#### Outcome effectiveness: meta-analysis

##### Fruit and vegetables

**Fig. 3:**
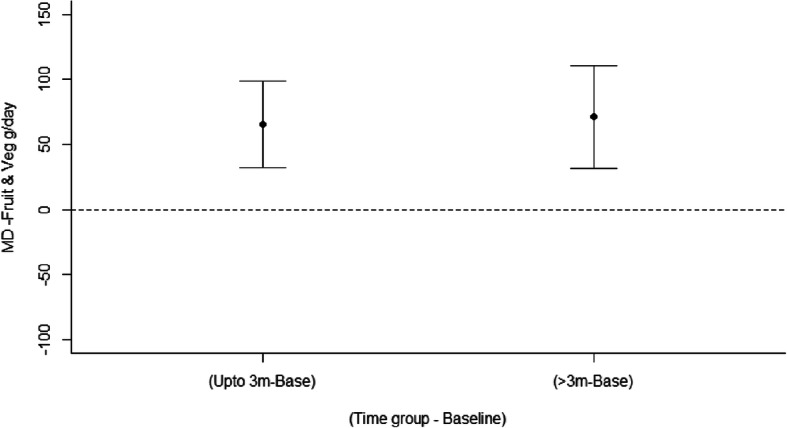
Mean differences for all interventions between intervention and control arms in fruit and vegetable intake (g/day) over time

Meta-analysis of change in fruit and vegetable intake (g/day) included 14 studies (12 gender-neutral and 2 gender-targeted) with a total of 19 intervention arms [28, 31, 34, 36, 44, 45, 47, 50–56] and examined two moderator effects. For all studies, there was a significant effect of time (LRT χ2(4) = 15.7, *p* < 0.001) with a significant mean increase in fruit and vegetable intake relative to baseline of + 65 g/day up to 3 months (95% CI: 32.7, 98.8) and + 71.2 g/day at > 3 months (95% CI: 31.6, 110.7) (Fig. 3). When compared to controls, there was a significant difference in change in fruit and vegetable intake over time between the gender-neutral and gender-targeted interventions (Wald χ^2^(2) = 6.69, *p* = 0.04). Specifically, when compared to controls, there was a significant mean increase in fruit and vegetable intake (g/day) in gender neutral studies; + 63.5 g/day up to 3 months (95% CI: 31.7, 95.2) and + 54.1 g/day for > 3 months (95% CI: 14.2, 94.1). For gender-targeted interventions there were minimal change up to 3 months − 1.1 g/day (95% CI: − 4.39, − 0.99), but a significant increase at > 3 months; + 158 g/day (95% CI: 64.7, 252.0). The funnel plot (Figure S2) symmetry indicated there was evidence of publication bias favouring studies with higher values, a nonparametric correlation test supported this (Kendall’s tau 0.23, *p* = 0.03). Plots of the means for the effects are in Figure S3, while model diagnostics are satisfactory and are in Figure S4. The forest plots showing mean difference (95% confidence interval) over time (months) are in Figure S5.

### Physical activity outcomes

Twenty-eight studies examined physical activity behaviours as the primary outcome (26%). A total of 20 studies were effective (71%). Most commonly, studies targeted time completing physical activity (*n* = 17, 61%), and of these, 10 studies were effective (59%).

Of the seven gender-targeted studies, six were effective (86%). Specifically, two studies each demonstrated significant differences in time completing physical activity [57, 58] and steps per day [59, 60], one study demonstrated significant differences in both time completing physical activity and steps per day [61], and one in gym attendance [62]. Pellitteri et al. found that females in an 8-week multicomponent intervention (weekly group sessions, website, weekly education materials) had a + 148 min per week and + 25 MET hours per week greater increase in PA compared with control participants (generic education materials) at post intervention [58]. In three studies, the utility of wearable activity trackers was compared with/without feedback or web-based components [57, 59, 61]. The interventions with additional components were more effective, with males reporting a greater increase in moderate-vigorous physical activity (MVPA) in one study at the end of the 12-week intervention (+2mins/week, *p* = 0.012) [57] and females reporting increased steps per day in two studies (+ 1972–3360 steps/day) at the end of a 4-week intervention and at 6-mon follow up after a 12-week intervention [59, 61]. In the study by Sriramatr et al. females also increased their leisure-time activity score [61]. Rote et al. found that an intervention with females to increase walking activity (self-tracking, feedback and updated goals) was enhanced by adding a Facebook social support group (+ 2636 steps/day compared with no Facebook group at the end of the 8-week intervention) [60]. Butryn et al. found that an intervention focusing on improving females attitudes and motivations towards PA resulted in + 0.58 days/week visiting the gym than a control group focusing on safe PA participation, assessed at the end of the 5-week intervention [62].

Of the 21 gender-neutral studies, 14 were effective (67%). Seven studies (54%) demonstrated significant changes in time completing PA [63–69], three and two studies demonstrated significant changes in exercise frequency [66, 70, 71] and steps per day respectively [72, 73], and one study each for gym attendance [74], meeting physical activity guidelines [75], and physical activity score [76]. Martens et al. reported + 0.7 days/week and + 35mins/week of completing vigorous PA at 1-month follow-up in participants who received a single session motivational intervention versus a standard educational brochure [66]. In studies by Bray et al. and Pfeffer et al. participants reported +78mins/week and + 0.86 h/week greater MVPA at 6-weeks and 1-week follow up respectively, following single session interventions which focused on action planning for PA versus no/alternative intervention control [63, 69]. Conner et al. and Cooke et al. found single session interventions involving affective messaging and self-affirmation around physical activity behaviours were effective compared with no intervention and non-affirmation at 3 and 1-week follow-up respectively, however actual change in time spent completing PA were not reported [64, 65]. Eisenberg et al. found higher self-reported PA in participants who recorded PA via an accelerometer versus those who were provided an accelerometer and/or e-diary and no intervention control (+ 1474–1756 MET minutes/week) at the end of the 1-week intervention [67]. Maselli et al. found greater increases in MVPA in participants of a goal setting and feedback intervention who were provided a wearable activity tracker compared with individual counselling sessions and no intervention control (+ 1311.4–2372.9 MET mins/week) at the end of the 12-week intervention [68]. The two studies by Wienstock et al. reported greater increases in days/week (+ 1.1 days/week, *p* = 0.001) and sessions/week exercising (+ 0.7sessions/week, *p* = 0.012) in intervention groups receiving motivational interviewing and motivational interviewing plus an exercise contract compared with exercise sessions alone or motivational interviewing plus contingency management, at the end of the 8-week interventions [70, 71]. Walsh et al. and Cooke et al. found that interventions involving tracking of steps per day were more effective when feedback or goal setting were included than tracking only (+ 1292–1310 steps/day), at the end of the 5 and 1-week interventions [72, 73]. Pope et al. reported greater achievement of gym attendance goals in participants who received continued monetary incentives to attend compared with discontinued and no incentive groups at the end of the 24-week intervention (+ 36% goals met, *p* < 0.001) [74]. Husband et al. reported a greater increase in PA score in participants who received face-to-face sessions focusing on self-identity and behaviour change techniques to increase PA compared with behaviour change techniques only at the end of the 2-week intervention (+ 4.5 points) [76]. Heeren et al. reported a greater likelihood of meeting PA guidelines at 12-month follow-up in participants in an 8-week health promotion intervention targeting PA, diet and alcohol delivered via group sessions, compared with one targeting HIV (OR = 3.35; 95% CI: 1.33–8.41) [75].

### Obesity outcomes

#### Outcome effectiveness: narrative summary

Thirty-six studies reported adiposity primary outcomes, including 18 weight gain prevention (50%) and 18 weight loss studies (50%). Of these 36 studies, 50% were effective (*n* = 18). Most commonly, studies targeted change in absolute weight (*n* = 29, 81%), and of these, 16 studies were effective (55%).

Among the 14 gender-targeted studies, five were effective (36%) [77–81], including three of nine weight gain prevention studies (33%) [78–80] and two of five weight loss studies (40%) [77, 81]. Four studies reported results in favour of the intervention group/s for absolute weight [77, 78, 80, 81] and one for absolute weight and BMI [79]. Two studies compared female targeted intervention programs focused on nutrition, PA and weight control against no intervention controls, with Eiben et al. reporting 4.5 kg greater weight loss at post intervention following a 12-month intervention [78], and Katterman et al. reporting 3.31 kg greater weight loss and − 1.08 kg/m^2^ greater change in BMI at 12 months following a 16-week intervention [79]. In the study by Tobias et al., of four intervention groups (weight loss manual, self-determination, behavioural contract and encouragement to lose weight of their own accord), females in the weight loss manual and behavioural contract groups achieved greater weight loss than no the intervention control (− 4.51 and − 4.97 pounds, *p* < 0.01) at 14-weeks following a 10-week intervention [81]. Ortega et al. compared two calorie deficit diets, one focusing on increased intake of grains and cereals and the other on increased vegetable intake, with females in the grains and cereals group achieving greater weight loss (− 0.8 kg) at the end of the 6-week intervention [77]. Klem et al. compared the same nutrition, PA and weight education program delivered via correspondence or via group sessions against a generic education control group with females, with the group format participants achieving greater weight loss compared with controls (− 1.7 kg, *p* < 0.05) at the end of the 10-week intervention [80].

Of the 22 gender-neutral studies, 13 were effective (59%), most of which were for absolute weight (*n* = 10, 59%). Six of nine weight gain prevention studies (67%) [82–87] and seven of 13 weight loss studies (54%) [88–94] were effective. Bertz et al. found that daily self-weighing with feedback achieved greater weight loss than irregular self-weighing without feedback over a one year intervention period (− 1.6 kg, *p* = 0.04) [82]. Two studies involved interventions focused on nutrition, PA and weight delivered in different face-to-face session formats [83, 90]. Hivert et al. reported greater changes in weight (− 1.3 kg, p = 0.04) and BMI (− 0.5 kg/m2, *p* = 0.01) for participants in the group session intervention compared with no intervention at the end of the 2-year intervention [83], while Phimarn et al. reported greater weight loss (− 1 kg, p = 0.04) for participants in the individual session intervention compared with group sessions at the end of the 6-month intervention [90]. LaRose et al. compared interventions targeting large changes/larger initial weight loss versus small changes/gradual weight loss, with greater weight loss achieved in the large changes group at 2 years (− 2 kg, *p* = 0.006) [84]. In two studies, e&mHealth interventions with more/less intervention components were compared. Napolitano et al. reported greater weight loss in participants in the intervention delivered via Facebook and text messaging with feedback, versus Facebook only and waitlist control at the end of the 8-week intervention (− 1.77–2.16 kg) [88], while Allman-Farinelli et al. reported a − 4.3 kg greater weight loss in participants in the enhanced mHealth intervention compared with controls (minimal version) at the end of the 9-month intervention [85]. Pearson et al. compared two telehealth weight management interventions; unscripted motivational interviewing or scripted education lessons, finding that the scripted lessons intervention participants achieved greater weight loss at the end of the 12 week intervention (− 5.26 kg, *p* = 0.05), however not sustained at 3 or 6 months [89]. Gow et al. compared three eHealth interventions (weekly weigh-in with feedback, weekly sessions covering nutrition, PA and body image, or both) with a no intervention control [86]. The group receiving both interventions achieved a greater reduction in BMI compared with the controls post the 6-week intervention (− 0.43 kg/m^2^, *p* < 0.05). Halperin et al. found a greater reduction in BMI in participants who received 10 weekly peer support sessions focusing on nutrition, physical activity and stress compared with usual care at 6-mon follow-up (− 2 kg/m^2^, *p* < 0.001) [94]. In the studies by Stephens et al. and Jakicic et al., standard weight loss interventions were compared with enhanced versions involving smartphone apps or wearable activity trackers for self-monitoring, with participants in the enhanced versions reporting greater reductions in weight (− 2.1–2.4 kg) at the end of the 12-week and 2-year interventions respectively [91, 92]. In the study by Stephens et al., intervention participants also reported greater reductions in BMI (− 1.2 kg/m2) and waist circumference (− 2.5 cm) [92]. Chiang et al. compared two physical activity focused interventions (12,000 steps/day goal and 12,000 steps/day goal with a faster rate of walking) with a no intervention control group, and reported a greater reduction in visceral fat area in the intervention group with a faster rate of walking compared with the other two groups at the end of the 8-week intervention (− 12.1–12.9%, *p* < 0.05) [93].

#### Outcome effectiveness: meta-analysis

##### Weight (kg) for weight gain prevention studies

**Fig. 4:**
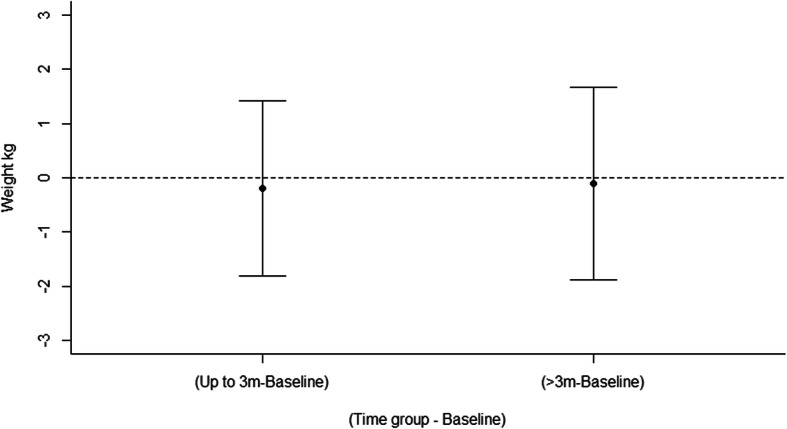
Mean differences for weight gain prevention interventions between intervention and control arms in weight (kg) over time

Meta-analysis of weight (kg) change outcomes included 7 studies (4 gender-neutral, 3 gender-targeted) with a total of 8 intervention arms [54, 95–97] for interventions to prevent weight gain and examined two moderator effects. There was no significant effect of time (LRT χ^2^(4) = 0.06, *p* = 0.969) with a non-significant mean change in weight relative to baseline − 0.20 kg up to 3 months (95% CI: − 1.81, 1.41) and − 0.10 kg for > 3 months (95% CI: − 1.88, 1.67) (Fig. 4). When compared to controls, there was no significant difference in weight change over time between the weight loss interventions and weight-gain prevention interventions (Wald χ^2^(2) = 0.10, *p* = 0.95). Specifically, when compared to controls, mean decrease in weight (kg) in gender-neutral interventions was − 1.34 kg up to 3 months (95% CI: − 2.66, − 0.03) and − 1.36 kg at > 3 months (95% CI: − 3.22, 0.50), and for gender-targeted interventions; − 2.77 kg up to 3 months (95% CI: − 6.18, 0.63) and − 2.16 kg at > 3 months (95% CI: − 4.35, 0.03). However, there were differences at baseline (with effect size treated as treatment – control) between gender-neutral interventions and control (− 1.25 kg, 95% CI: − 2.44, − 0.06) and between gender –targeted studies and controls (− 2.00 kg, 95% CI: − 4.04, 0.05) which attenuates the estimated changes at up to 3 months and beyond (Supporting information, Figure S6). The funnel plot (Figure S7) demonstrated symmetry, indicating there was evidence of publication bias favouring studies with higher values, a nonparametric correlation test supported this (Kendall’s tau 0.37, *p* = 0.03). Plots of the means for the effects are in Figure S8, while model diagnostics are satisfactory and are in Figure S9. The forest plots showing mean difference (95% confidence interval) over time (months) are in Figure S10.

##### Weight (kg) for weight loss studies

**Fig. 5:**
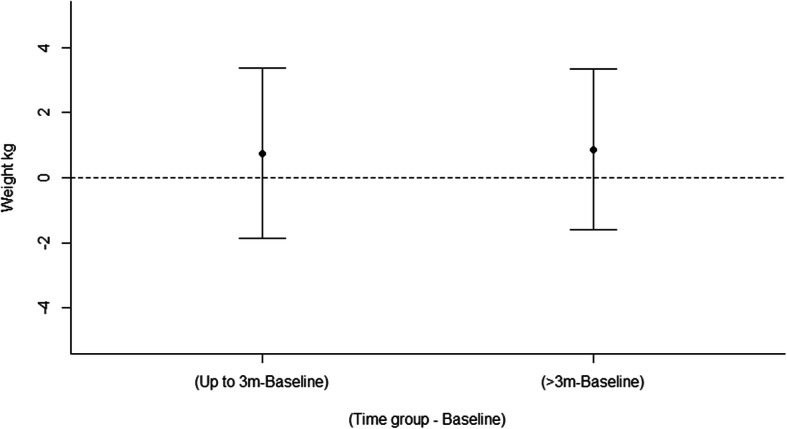
Mean differences for weight loss interventions between intervention and control arms in weight (kg) over time

Meta-analysis of weight (kg) change outcomes included 7 studies (4 gender-neutral, 3 gender-targeted) with a total of 8 intervention arms [77, 89, 93, 98–101] for weight loss interventions and examined two moderator effects. There was no significant effect of time (LRT χ^2^(4) = 1.74, *p* = 0.418) with a non-significant mean change in weight relative to baseline + 0.75 kg up to 3 months (95% CI: − 1.87, 3.38) and + 0.87 kg at > 3 months (95% CI: − 1.60, 3.34) (Fig. 5). When compared to controls, there was no significant difference in weight change over time between gender-neutral and gender-targeted interventions (Wald χ^2^(2) = 3.20, *p* = 0.20). Specifically, when compared to controls, mean decrease in weight (kg) in gender-neutral interventions were − 2.06 kg up to 3 months (95% CI: − 6.45, 2.35) and − 1.70 kg at > 3 months (95% CI: − 5.31, 1.91), and for gender-targeted interventions; − 2.61 kg up to 3 months (95% CI: − 6.07, 0.84) and − 0.28 kg for > 3 months (95% CI: − 5.40, 4.84). However, there were differences at baseline (with effect size treated as treatment – control) between gender-neutral interventions and controls (− 1.27 kg, 95% CI: − 4.93, 2.39) and between gender–targeted studies and controls (− 5.00 kg, 95% CI: − 8.87, − 1.12) which attenuated the estimated changes at up to 3 months and beyond (Supporting information, Figure S11). The funnel plot (Figure S12) demonstrated no symmetry, indicating there was no evidence of publication bias favouring studies with higher values, a nonparametric correlation test supported this (Kendall’s tau − 0.14, *p* = 0.37). Plots of the means for the effects are in Figure S13, while model diagnostics are satisfactory and are in Figure S14. The forest plots showing mean difference (95% confidence interval) over time (months) are in Figure S15.

### Nutrition and physical activity outcomes

Six studies reported nutrition and physical activity related primary outcomes, of which five were effective for one or both outcomes (83%) [55, 56, 102–104]. Of the six studies, one was male targeted, although no significant between group differences were reported [105]. All five studies which were effective were gender-neutral studies [55, 56, 102–104]. Two studies each reported significant differences in exercise frequency [56, 104], fruit and/or vegetable intake [103, 104], and percentage energy intake from fat [55, 104]. One study each reported significant differences in meeting PA guidelines [103] and time spent completing PA [102].

In two studies, single session interventions were more effective than no intervention control or generic education control [56, 104]. These involved motivational interviewing and action planning [104], or a goal setting consultation [56], and reported greater changes in percent energy intake from fat (− 4.1%), fruit and vegetable intake (+ 0.8serves/day, *p* = 0.02) and physical activity (+ 0.5 days/week, *p* < 0.01) at 4-week follow-up [104], or a greater increase in PA score (reflecting more days/week of PA) at 12-mon follow-up respectively [56]. Schweitzer et al. reported a greater reduction in saturated fat intake (− 1.3%, *p* = 0.048) in participants who received tailored diet and PA goals via email compared with controls (fact sheets on alternative health behaviours) at the end of the 6-mon intervention [55]. In the studies by Kypri et al. and Sandrick et al., assessment of baseline diet and PA behaviours was enhanced by receiving feedback or feedback and ongoing text message support respectively [102, 103]. Kypri reported that a greater proportion of participants met recommendations for fruit and vegetable intake (33% vs 13%, p = 0.02) and PA (90% vs 71%) at 6-weeks following the single session intervention [103], while Sandrick reported + 646 MET mins/week of PA at the end of the 8-week intervention [102], in the enhanced groups.

### Obesity, nutrition and physical activity outcomes

Overall, five studies reported adiposity, nutrition and physical activity as primary outcomes, and three of these were effective for one or more outcomes (60%) [50, 51, 106]. All were weight gain prevention studies. One of the five studies was female targeted, however no significant between group differences were reported [107]. All three of the effective studies were gender-neutral studies. All three reported significant differences in fruit and/or vegetable intake [50, 51, 106], with one also reporting a significant difference in time completing physical activity [50].

Kattelman et al. reported a greater increase in FV intake (+ 0.4cups/day) in participants who received weekly face-to-face education plus email nudges over 10 weeks compared with waitlist controls at program end, however not sustained at 15 month follow-up [51]. LaChausse et al. and Greene et al. found that eHealth nutrition and PA programs were more effective compared with face-to-face delivery and/or no intervention control [50, 106]. LaChausse reported greater increases in fruit and vegetable intake (+ 0.7–0.8 serves/day fruit and + 0.21–0.22 serves/day veg) at 14-week follow-up after a 12-week intervention [106], and Greene et al. reported greater increases in fruit and vegetable intake (+ 0.5cups/day, *p* < 0.001) and PA (+ 270 MET-min/wk) (*p* < 0.05) at 15-month follow-up after a 10-week intervention [50].

### Obesity and physical activity outcomes

Three studies reported adiposity and physical activity as primary outcomes, two were gender-neutral weight gain prevention studies [108, 109] and one female targeted weight loss study [101]. One gender-neutral study was effective, reporting a greater increase in the time spent completing physical activity (+ 1545.8 MET mins/week, *p* < 0.001) in participants who received nutrition education delivered via three modes compared with a no intervention control group, at the end of the 10-week intervention [108].

## Discussion

This is the first review to summarise the outcome effectiveness of interventions targeting nutrition, physical activity or overweight/obesity in young adults by gender-targeted versus gender-neutral interventions, and the largest review to date in this area. Studies were predominantly gender-neutral, and gender-targeted studies were predominantly in females. Comparison of outcome effectiveness by chi-square tests including all studies found no statistically significant differences between gender-targeted and gender-neutral studies. Although not significant, a greater proportion of gender-targeted studies were effective in improving nutrition and physical activity outcomes, and a greater proportion of gender-neutral studies were effective for adiposity outcomes. Meta-analyses for weight outcomes found no significant differences between gender-targeted and gender-neutral studies, including for weight loss and weight gain prevention studies. Meta-analysis of fruit and vegetable intakes demonstrated significant differences between gender-neutral and gender-targeted studies, with intervention participants in gender-neutral studies significantly increasing intake by + 63.5 g/day up to 3 months compared to controls, while participants in gender-targeted studies demonstrated a significantly greater increase of + 158 g/day for > 3 months. However, this finding should be interpreted with caution due to including only two gender-targeted studies in the meta-analysis. The findings collectively are suggestive of a potential difference between gender-targeted and gender-neutral interventions for young adults.

### Effectiveness of gender-targeted versus gender-neutral interventions targeting nutrition, physical activity or overweight/obesity

In the current review, a higher percentage of interventions targeting nutrition only and physical activity only were effective among gender-targeted compared with gender-neutral studies. However, a higher percentage of interventions targeting overweight/obesity were effective in gender-neutral compared with gender-targeted studies, including both weight gain prevention and weight loss interventions. Although these findings were not significant they indicate that for the individual behaviours, diet and physical activity, a gender-targeted approach may be more effective, however for weight gain prevention and weight loss, this is not the case at this stage. The meta-analysis results support these findings, with a significantly greater increase in fruit and vegetable intake identified for gender-targeted studies at follow up > 3 months, and no differences in weight identified between gender-neutral and gender-targeted interventions for weight loss or weight gain prevention studies. However, there were differences in the proportion of interventions that were weight loss and weight gain prevention between gender-neutral and gender-targeted studies, as well as a higher mean intervention duration in gender-neutral studies, which may help to explain the differences in effectiveness seen. Additionally, the meta-analysis for fruit and vegetable intake included only two gender-targeted studies, and therefore these results are preliminary. Overall, the findings suggest that a gender-targeted approach may be more effective in the case of nutrition and physical activity behaviours, while further studies are needed to determine differences for all outcomes.

The findings of potential differences between gender-targeted and gender-neutral studies are in line with the evidence that males and females have different motivations and barriers for healthy eating and physical activity, and different intervention preferences [16, 18, 110, 111], which a targeted intervention can address. Another factor to consider in the gender-targeted versus gender-neutral intervention debate is gender norms, or the underlying societal rules of acceptable behaviours, attitudes and image for men and women [112]. Gender normative influences begin early in life and recent explorations of global survey data suggest that these are often context-specific (e.g. different cultures) and that, intertwined with other social factors, they can impact on health outcomes across the life course [112]. Individuals are inclined to engage in health behaviours and strive for an image and identity that aligns with gender norms, and this may influence their engagement in and outcomes of a health behaviour intervention. Beyond targeting of interventions, it is also evidenced that interventions which are tailored, that is, consider personal characteristics and mediators of behaviour [113], may be more engaging and effective. However, in this review, only five gender-targeted studies were tailored, which may also explain some of the lack of effectiveness found. Tailoring included for example, considering participants interests, preferences, circumstances, stage of change, motivations and/or barriers in regards to intervention content and/or delivery [101, 105, 107, 114, 115].

In terms of the types of interventions that demonstrated effectiveness in this review, there was consistency between gender-targeted and gender-neutral studies. Interventions which involved instructional elements, such as feedback, active participation, such as goal setting or action planning, and supportive elements, such as motivational interviewing or social support networks, seemed to be more effective than those without, particularly where these were tailored. Further, interventions involving generic education only were largely ineffective. These findings are consistent with previous reviews of health behaviour interventions in adults more broadly, including interventions targeting dietary and physical activity behaviours [116, 117]. This is therefore additive to the evidence that in order to effectively change behaviour, interventions need to provide actionable content, incorporate differentiation to achieve relevance on an individual level, and offer means of support for individuals to change their behaviour [116–118]. Other factors to consider in developing effective interventions, and that may differ between gender-targeted and gender-neutral interventions, include the framing of intervention content and messages, the motivations/barriers that are focused on or highlighted, and the mode of intervention delivery [110, 119].

### Strengths and limitations of the included studies

The risk of bias assessment identified mainly limitations of the included studies. Generally, studies provided sufficient detail with regards to study attrition and any exclusions from the analysis. However, the majority of studies lacked sufficient detail to assess the risk of bias in terms of selective outcome reporting, blinding, and allocation concealment. In terms of the study samples, these predominantly included female participants. As such, there was not enough gender-targeted interventions to explore whether male or female targeted interventions are more effective compared with gender-neutral. Further, there was a large variation in studies’ reporting of outcomes in terms of units reported, and some studies failed to report values for all timepoints, which limits the comparability between studies. The measurement methods used for individual outcomes across studies were predominantly objective for adiposity, self-report for nutrition and approximately 50% objective/50% self-report for physical activity. The potential for bias from self-report outcomes should be considered when interpreting the review findings, in particular for nutrition outcomes.

### Strengths and limitations of the review

The major strengths of this review include that it is the largest and most comprehensive review in this research area, and the assessment of differences between gender-targeted and gender-neutral interventions. Exploring outcome effectiveness by gender-targeted and gender-neutral interventions gives a more detailed perspective to try and understand intervention effectiveness. The fact that there was a fairly even spread of studies with the primary outcome/s being nutrition, physical activity or adiposity outcomes is also a strength, as it gives more validity to the comparisons made between studies. Further, a comprehensive search strategy was used for the review. The inclusion of the meta-analysis for weight and fruit and vegetable intake further strengthens the review findings. However, the results for fruit and vegetable intake should be interpreted with caution due to the inclusion of only two gender-targeted studies. For most outcomes, meta-analysis was not able to be conducted due to an insufficient number of studies reporting outcomes in adequate detail. This includes no meta-analysis for physical activity outcomes. In terms of limitations, the definition of effectiveness used has limitations in that it is a binary classification. For example, some studies were classified as not effective on the basis that results for primary outcomes were not significant, however results for secondary outcome/s were significant. Additionally, this review focuses on one aspect that may contribute to intervention effectiveness, however there are many factors that could play a role. Studies were also limited to those published in English, which may have excluded relevant studies and could limit the generalisability of the findings.

### Recommendations from this research

To truly determine the effectiveness of gender-targeted interventions versus gender-neutral interventions there needs to be well-designed RCTs that specifically compare the two intervention approaches, and control for all other factors that may influence outcome effectiveness (e.g. intervention duration, behaviour change techniques used) [120]. Further to this, studies should explore factors that may mediate effectiveness in gender-targeted and gender-neutral interventions (e.g. gender-tailored or non-gendered content, behaviour change techniques used and barriers to achieving a healthy lifestyle). Given the increasing diversity of gender identities within society, and in particular in young adults, it would also be pertinent to explore whether gender identity is a mediating factor in intervention effectiveness.

To improve the methodological quality and reporting of RCTs in this area, studies should:

- Clearly describe primary and secondary outcomes to allow for easier interpretation of effectiveness and comparability between studies.
- Report outcomes more consistently in terms of units to allow for meta-analysis (e.g. grams of fruit and vegetable, minutes of physical activity).
- Use a behaviour change technique checklist to report the techniques included within interventions, to help identify and rigorously compare similar combinations of techniques [21].
- Adhere to the CONSORT guidelines so that the methods undertaken are clear and transparent, and risk of bias can be appropriately assessed.
- Recruit more diverse samples of young adults from different settings as most of the study populations included in this review were female, aged 17–25 years, and in the University setting.

## Conclusions

The current review found no statistically significant differences between gender-targeted and gender-neutral studies for overall outcome effectiveness. Although not significant, a greater proportion of gender-targeted studies were effective in improving nutrition and physical activity outcomes, and a greater proportion of gender-neutral studies were effective for adiposity outcomes. The lack of significance is likely due to an insufficient number of studies to detect a difference. The meta-analysis results found a significantly greater increase in fruit and vegetable intake for participants of gender-targeted studies at follow up > 3 months, however no differences in weight between gender-neutral and gender-targeted studies for weight loss or weight gain prevention studies. The findings indicate a potential difference between gender-targeted and gender-neutral studies, which warrants further investigation. A key recommendation is for more detailed and standardised reporting of study details, particularly outcome measures, to facilitate future efforts to determine the most effective intervention strategies for changing young adults’ diet and physical activity practices and adiposity.

## Supplementary information

**Additional file 1 **: **Table S1.** Search Terms by database. **Table S2.** Detailed study characteristics of included studies - Gender-targeted studies (*n* = 30). **Table S3.** Detailed study characteristics of included studies - Gender-neutral studies (*n* = 77). **Figure S1.** Mean differences by gender-neutral or gender targeted interventions and control arms in fruit and vegetable intake (g/day) over time. **Figure S2.** Funnel plot vs Standard Error – Fruit and vegetables (g/day). **Figure S3.** Plots of the means for effect – Fruit and vegetables (g/day). **Figure S4.** Model diagnostics – Fruit and vegetables (g/day). **Figure S5.** Forest plot – Fruit and vegetable intake (g/day). **Figure S6.** Mean differences by gender-neutral or gender targeted interventions and control arms in weight (kg) over time among weight gain prevention interventions. **Figure S7.** Funnel plot vs Standard Error – Weight (kg) in weight gain prevention interventions. **Figure S8.** Plots of the means for effect – Weight (kg) in weight gain prevention interventions. **Figure S9.** Model diagnostics – Weight (kg) in weight gain prevention interventions. **Figure S10.** Forest plot – Weight (kg) in weight gain prevention interventions. **Figure S11.** Mean differences by gender-neutral or gender targeted interventions and control arms in weight (kg) over time among weight loss studies. **Figure S12.** Funnel plot vs Standard Error – Weight (kg) in weight loss studies. **Figure S13.** Plots of the means for effect – Weight (kg) in weight loss studies. **Figure S14.** Model diagnostics – Weight (kg) in weight loss studies. **Figure S15. F**orest plot – Weight (kg) in weight loss studies.

## Data Availability

Not applicable.

## Abbreviations

RCT: Randomised controlled trial
BMI: Body mass index
MVPA: Moderate-vigorous physical activity
