# Correction to: Effectiveness of gender-targeted versus gender-neutral interventions aimed at improving dietary intake, physical activity and/or overweight/obesity in young adults (aged 17–35 years): a systematic review and meta-analysis

**DOI:** 10.1186/s12937-020-00605-0

**Published:** 2020-08-26

**Authors:** Thomas Sharkey, Megan C. Whatnall, Melinda J. Hutchesson, Rebecca L. Haslam, Aaron Bezzina, Clare E. Collins, Lee M. Ashton

**Affiliations:** 1 School of Health Sciences, Faculty of Health and Medicine, University of Newcastle, Callaghan, 2308 Australia; 2 Priority Research Centre for Physical Activity and Nutrition, University of Newcastle, Callaghan, 2308 Australia

**Correction to: Nutr J 19, 78 (2020)**

**https://doi.org/10.1186/s12937-020-00594-0**

Following publication of the original article [1], the authors would like to correct the mix up in intervention groups when reporting results of one paper in the third paragraph under the heading **Physical activity outcomes**.

The sentence currently reads:

Maselli et al. found greater increases in MVPA in participants of a goal setting and feedback intervention who were provided a wearable activity tracker compared with individual counselling sessions and no intervention control (+ 1311.4–2372.9 MET mins/week)

The sentence should read:

Maselli et al. found greater increases in MVPA in participants who were provided with individual counselling sessions compared to a goal setting and feedback intervention who were provided a wearable activity tracker and a no intervention control group (+ 1311.4–2372.9 MET mins/week)
