# Supplementary material for: Effectiveness of gender-targeted versus gender-neutral interventions aimed at improving dietary intake, physical activity and/or overweight/obesity in young adults (aged 17–35 years): a systematic review and meta-analysis
Source: Nutr J. 2020 Jul 30;19:78. doi: 10.1186/s12937-020-00594-0 (PMC7393713; doi:10.1186/s12937-020-00594-0)
Supplement: Supplementary file 1 — Additional file 1 : Table S1. Search Terms by database. Table S2. Detailed study characteristics of included studies - Gender-targeted studies (n = 30). Table S3. Detailed study characteristics of included studies - Gender-neutral studies (n = 77). Figure S1. Mean differences by gender-neutral or gender targeted interventions and control arms in fruit and vegetable intake (g/day) over time. Figure S2. Funnel plot vs Standard Error – Fruit and vegetables (g/day). Figure S3. Plots of the means for effect – Fruit and vegetables (g/day). Figure S4. Model diagnostics – Fruit and vegetables (g/day). Figure S5. Forest plot – Fruit and vegetable intake (g/day). Figure S6. Mean differences by gender-neutral or gender targeted interventions and control arms in weight (kg) over time among weight gain prevention interventions. Figure S7. Funnel plot vs Standard Error – Weight (kg) in weight gain prevention interventions. Figure S8. Plots of the means for effect – Weight (kg) in weight gain prevention interventions. Figure S9. Model diagnostics – Weight (kg) in weight gain prevention interventions. Figure S10. Forest plot – Weight (kg) in weight gain prevention interventions. Figure S11. Mean differences by gender-neutral or gender targeted interventions and control arms in weight (kg) over time among weight loss studies. Figure S12. Funnel plot vs Standard Error – Weight (kg) in weight loss studies. Figure S13. Plots of the means for effect – Weight (kg) in weight loss studies. Figure S14. Model diagnostics – Weight (kg) in weight loss studies. Figure S15. Forest plot – Weight (kg) in weight loss studies. [file 12937_2020_594_MOESM1_ESM.docx]

Supporting information

Effectiveness of gender targeted versus gender-neutral interventions aimed at improving dietary intake, physical activity and/or overweight/obesity in young adults (aged 17-35 years): a systematic review and meta-analysis

Thomas Sharkey, Megan C. Whatnall, Melinda J. Hutchesson, Rebecca L. Haslam, Aaron Bezzina, Clare E. Collins, and Lee M. Ashton ^1,2*^

***** Correspondence: lee.ashton@newcastle.edu.au; Tel.: +61-2-49-138034

**Contents**

| **Supporting information item** | **Page** |
| --- | --- |
| Table S1: Search Terms by database | 2 |
| Table S2: Detailed study characteristics of included studies - Gender-targeted studies (n=30) | 8 |
| Table S3: Detailed study characteristics of included studies - Gender-neutral studies (n=77) | 19 |
| Figure S1: Mean differences by gender-neutral or gender targeted interventions and control arms in fruit and vegetable intake (g/day) over time. | 47 |
| Figure S2: Funnel plot vs Standard Error – Fruit and Vegetables (g/day) | 47 |
| Figure S3: Plots of the means for effect – Fruit and Vegetables (g/day) | 48 |
| Figure S4: Model diagnostics – Fruit and Vegetables (g/day) | 48 |
| Figure S5: Forest plot – fruit and vegetable intake (g/day) | 49 |
| Figure S6: Mean differences by gender-neutral or gender targeted interventions and control arms in weight (kg) over time among weight gain prevention interventions. | 50 |
| Figure S7: Funnel plot vs Standard Error – Weight (kg) in weight gain prevention interventions. | 50 |
| Figure S8: Plots of the means for effect – Weight (kg) in weight gain prevention interventions. | 51 |
| Figure S9: Model diagnostics – Weight (kg) in weight gain prevention interventions. | 51 |
| Figure S10: Forest plot – weight (kg) in weight gain prevention interventions. | 52 |
| Figure S11: Mean differences by gender-neutral or gender targeted interventions and control arms in weight (kg) over time among weight loss studies | 53 |
| Figure S12: Funnel plot vs Standard Error – Weight (kg) in weight loss studies | 53 |
| Figure S13: Plots of the means for effect – Weight (kg) in weight loss studies | 54 |
| Figure S14: Model diagnostics – Weight (kg) in weight loss studies | 54 |
| Figure S15: Forest plot – weight (kg) in weight loss studies | 55 |

**Table S1:** Search Terms by database

*Medline*

| **#** | **Searches** |
| --- | --- |
| 1 | randomized controlled trial/ |
| 2 | controlled Clinical Trial/ |
| 3 | random*.tw. |
| 4 | trial.tw. |
| 5 | (control adj group*).tw. |
| 6 | 1 or 2 or 3 or 4 or 5 |
| 7 | (young* adj (adult* or men or male* or man or women or female* or woman)).tw. |
| 8 | Young Adult/ |
| 9 | college aged.tw. |
| 10 | ((university or college*) adj student*).tw. |
| 11 | 7 or 8 or 9 or 10 |
| 12 | Diet/ |
| 13 | Healthy Diet/ |
| 14 | eating/ |
| 15 | eating behavio?r.tw. |
| 16 | physical activity/ |
| 17 | Exercise/ |
| 18 | Weight Loss/ |
| 19 | obesity/ or obesity, abdominal/ or obesity, metabolically benign/ or obesity, morbid/ |
| 20 | Overweight/ |
| 21 | 12 or 13 or 14 or 15 or 16 or 17 or 18 or 19 or 20 |
| 22 | 6 and 11 and 21 |
| 23 | animals/ not (humans/ and animals/) |
| 24 | 22 not 23 |
| 25 | addresses/ or lectures/ or anecdotes/ or biography/ or interview/ or comment/ or directory/ or editorial/ or legal cases/ or case reports/ or legislation/ or letter/ or news/ or newspaper article/ or patient education handout/ |
| 26 | 24 not 25 |
| 27 | limit 26 to english language |

*Embase*

| **#** | **Searches** |
| --- | --- |
| 1 | randomized controlled trial/ |
| 2 | controlled Clinical Trial/ |
| 3 | random*.tw. |
| 4 | trial.tw. |
| 5 | (control adj group*).tw. |
| 6 | 1 or 2 or 3 or 4 or 5 |
| 7 | (young* adj (adult* or men or male* or man or women or female* or woman)).tw. |
| 8 | Young Adult/ |
| 9 | college aged.tw. |
| 10 | ((university or college*) adj student*).tw. |
| 11 | 7 or 8 or 9 or 10 |
| 12 | diet/ |
| 13 | healthy diet/ |
| 14 | dietary intake/ |
| 15 | eating/ |
| 16 | eating behavio?r.tw. |
| 17 | physical activity/ |
| 18 | exercise/ |
| 19 | obesity/ or abdominal obesity/ or metabolically benign obesity/ or morbid obesity/ |
| 20 | weight reduction/ |
| 21 | 12 or 13 or 14 or 15 or 16 or 17 or 18 or 19 or 20 |
| 22 | 6 and 11 and 21 |
| 23 | animals/ not (humans/ and animals/) |
| 24 | 22 not 23 |
| 25 | addresses/ or lectures/ or anecdotes/ or biography/ or interview/ or comment/ or directory/ or editorial/ or legal cases/ or case reports/ or legislation/ or letter/ or news/ or newspaper article/ or patient education handout/ |
| 26 | 24 not 25 |
| 27 | limit 26 to english language |

*PsycINFO*

| **#** | **Searches** |
| --- | --- |
| 1 | clinical trials/ |
| 2 | random*.tw. |
| 3 | trial.tw. |
| 4 | (control adj group*).tw. |
| 5 | 1 or 2 or 3 or 4 |
| 6 | (young* adj (adult* or men or male* or man or women or female* or woman)).tw. |
| 7 | college aged.tw. |
| 8 | ((university or college*) adj student*).tw. |
| 9 | 6 or 7 or 8 |
| 10 | diets/ |
| 11 | eating behavior/ or food intake/ |
| 12 | physical activity/ or active living/ or activity level/ |
| 13 | EXERCISE/ |
| 14 | weight loss/ or weight control/ |
| 15 | obesity/ or overweight/ |
| 16 | 10 or 11 or 12 or 13 or 14 or 15 |
| 17 | 5 and 9 and 16 |
| 18 | animals/ not (humans/ and animals/) |
| 19 | 17 not 18 |
| 20 | addresses/ or lectures/ or anecdotes/ or biography/ or interview/ or comment/ or directory/ or editorial/ or legal cases/ or case reports/ or legislation/ or letter/ or news/ or newspaper article/ or patient education handout/ |
| 21 | 19 not 20 |
| 22 | limit 21 to english language |

*Web of Science*

| **#** | **Searches** |
| --- | --- |
| # 15 | (#14 AND #10 AND #5) *AND***LANGUAGE:** (English) *AND* **DOCUMENT TYPES:** (Article OR Review)  *Indexes=SCI-EXPANDED, SSCI, A&HCI, ESCI Timespan=All years* |
| # 14 | #13 OR #12 OR #11  *Indexes=SCI-EXPANDED, SSCI, A&HCI, ESCI Timespan=All years* |
| # 13 | TS=("Exercise" or "Physical activity")  *Indexes=SCI-EXPANDED, SSCI, A&HCI, ESCI Timespan=All years* |
| # 12 | TS=("Diet" or "nutrition" or "healthy diet" or "eating habits" "eating behavi?r")  *Indexes=SCI-EXPANDED, SSCI, A&HCI, ESCI Timespan=All years* |
| # 11 | TS= ("Obesity" or "overweight" or "obesity, abdominal", or "weight loss" or "obesity, morbid")  *Indexes=SCI-EXPANDED, SSCI, A&HCI, ESCI Timespan=All years* |
| # 10 | #9 OR #8 OR #7 OR #6  *Indexes=SCI-EXPANDED, SSCI, A&HCI, ESCI Timespan=All years* |
| # 9 | TS= ("young adult*")  *Indexes=SCI-EXPANDED, SSCI, A&HCI, ESCI Timespan=All years* |
| # 8 | TS= ("university student*" or "college student*").  *Indexes=SCI-EXPANDED, SSCI, A&HCI, ESCI Timespan=All years* |
| # 7 | TS= ("college aged" or "student*")  *Indexes=SCI-EXPANDED, SSCI, A&HCI, ESCI Timespan=All years* |
| # 6 | TS= ("young men" or "young male*" or "young man" or "young women" or "young female*" or "young woman")  *Indexes=SCI-EXPANDED, SSCI, A&HCI, ESCI Timespan=All years* |
| # 5 | #1 or #2 or #3 or #4  *Indexes=SCI-EXPANDED, SSCI, A&HCI, ESCI Timespan=All years* |
| # 4 | TS=("control group*") OR TS=("trial" or "clinical trial" or "clinical trials" or "controlled trial" or "controlled trials")  *Indexes=SCI-EXPANDED, SSCI, A&HCI, ESCI Timespan=All years* |
| # 3 | TS=("random*")  *Indexes=SCI-EXPANDED, SSCI, A&HCI, ESCI Timespan=All years* |
| # 2 | TS= ("controlled clinical trial")  *Indexes=SCI-EXPANDED, SSCI, A&HCI, ESCI Timespan=All years* |
| # 1 | TS=("randomized controlled trial")  *Indexes=SCI-EXPANDED, SSCI, A&HCI, ESCI Timespan=All years* |

*Cochrane*

| **ID** | **Search** |
| --- | --- |
| #1 | MeSH descriptor: [Controlled Clinical Trials as Topic] this term only |
| #2 | MeSH descriptor: [Randomized Controlled Trials as Topic] this term only |
| #3 | MeSH descriptor: [Randomized Controlled Trial] explode all trees |
| #4 | (random*):ti,ab,kw |
| #5 | (trial):ti,ab,kw |
| #6 | (control next group*):ti,ab,kw |
| #7 | (#1 or #2 or #3 or #4 or #5 or #6) |
| #8 | MeSH descriptor: [Young Adult] this term only |
| #9 | (young* next (adult* or men or male* or man or women or female* or woman)):ti,ab,kw |
| #10 | (college aged):ti,ab,kw |
| #11 | ((university or college*) next student*):ti,ab,kw |
| #12 | #8 or #9 or #10 or #11 |
| #13 | MeSH descriptor: [Diet] explode all trees |
| #14 | MeSH descriptor: [Healthy Diet] this term only |
| #15 | (eating behavio?r):ti,ab,kw |
| #16 | MeSH descriptor: [Exercise] this term only |
| #17 | MeSH descriptor: [Weight Loss] this term only |
| #18 | MeSH descriptor: [Obesity] 1 tree(s) exploded |
| #19 | MeSH descriptor: [Overweight] this term only |
| #20 | (#13 or #14 or #15 or #16 or #18 or #19) |
| #21 | (#7 and #12 and #20) |
| #22 | (addresses):pt or (lectures):pt or (anecdotes):pt or (biography):pt or (interview):pt or (comment):pt or (directory):pt or (editorial):pt or (legal cases):pt or (case reports):pt or (legislation):pt or (letter):pt or (news):pt or (newspaper article):pt or (patient education handout):pt |
| #23 | (#21 not #22) |

*Cinahl*

| **#** | **Search** |
| --- | --- |
| S20 | S6 AND S12 AND S19 |
| S19 | S13 OR S14 OR S15 OR S16 OR S17 OR S18 |
| S18 | (MH "Weight Loss") |
| S17 | (MH "Obesity") OR (MH "Obesity, Morbid") |
| S16 | (MH "Exercise") |
| S15 | (MH "Physical Activity") |
| S14 | (MH "Nutrition") |
| S13 | (MH "Diet+") |
| S12 | S7 OR S8 OR S9 OR S10 OR S11 |
| S11 | TI ((university or college*) N1 student*) or AB ((university or college*) N1 student*) |
| S10 | TI "college aged" OR AB "college aged" |
| S9 | (MH "Students, College") OR (MH "Students, Undergraduate") OR (MH "Students, Graduate") |
| S8 | (MH "Young Adult") |
| S7 | TI ( (young* N1 (adult* or men or male* or man or women or female or woman)) ) OR AB ( (young* N1 (adult* or men or male* or man or women or female or woman)) ) |
| S6 | S1 OR S2 OR S3 OR S4 OR S5 |
| S5 | TI (control N1 group*) OR AB (control N1 group*) |
| S4 | TI (trial) OR AB (trial) |
| S3 | TI (random*) OR AB (random*) |
| S2 | (MH “randomized controlled trials”) |
| S1 | (MH "clinical trials") |

**Table S2:** Detailed study characteristics of included studies - Gender-targeted studies (n=30)

| **First author, year, study, name, citation** | **Country** | **Study participants (age range; % male/female; ethnicity)** | **N at baseline** | **Intervention (I)/ Comparator (C)** | **Intervention Focus (Nutrition/Physical activity/ Obesity)** | **Delivery Mode** | **Intervention Duration (weeks)** | **Recruitment setting** | **Retention** | **Data collection timepoints** | **Primary outcome measure/s (measurement method)** | **Effect (between group difference)** |
| --- | --- | --- | --- | --- | --- | --- | --- | --- | --- | --- | --- | --- |
| Ashton, 2017, HEYMAN, [1] | Australia | 17- ≤25yrs; 100% male; NR | 50 | I: Access to website, F2F (group and individual), jawbone wearable device, portion control tool, gymstick resistance band, personalised food and nutrient report; a private facebook discussion group C: Waitlist control | Nutrition and Physical Activity + well-being | F2F, website, social media | 12 | Community | 3-mon: 94% | 0, 3-m | Steps per day (pedometer) Diet quality score (self-report FFQ) | No sig between grp difference in Diet quality (ARFS total score) +3.6 (−0.4, 7.6, p=0.08) or Steps +1012.7 (−506.2, 2531.6, p=0.191) at 3-mon. |
| Cambien, 1981, Paris Cardiovascular Risk Factor Prevention Trial, [2] | France | 18- ≤35yrs; 100% male; NR | 3336 | I: 4x sessions with the doctor who provided advice on diet, PA & smoking; also provided with printed docs to support behaviours. C: No information on control group. | Nutrition and Physical Activity + smoking | F2F + print materials | 52 | Workplace | NR | 0, 2 yrs | Weight (kg) (measured) | No significant between group difference for weight at 2-yr. Within-grp changes: INT grp +0.8 (SD3.4) and CONTROL +1.2 (SD 3.4). |
| Middleton, 2014, [3] | USA | 18- ≤25yrs; 100% female; predominantly white | 95 | I: 5 session weight prevention program + online self-monitoring, key components included decreasing calories and increasing PA. C: Wait list | Nutrition, Physical Activity and Obesity | F2F + online | 4 | University | NR | 0, 7, 15 wks | Weight (kg) (measured) | Weight loss between groups not significant at post test (Intervention group mean wt loss of -2.07 ± 2.52 kg) or FU (Intervention group net change of −1.02± 2.61 kg from baseline) |
| Pellitteri, 2017, [4] | USA | 18- ≤25yrs; 100% female; predominantly white | 37 | I: Program: Fit-Minded College Edition. Weekly meetings, given 3 issues of a womens health magazine + website with online workbook, and a syllabus outlining weekly readings/ activities. C: 3 issues of a womens health magazine | Physical Activity  + self-worth | F2F + print materials + website | 8 | University | 8-wks: 73% | 0, 8 weeks | Leisure time PA and MET hours per week (self-report questionnaire) | Significant difference favouring intervention group for mins/wk of leisure time PA (Int: +169mins/wk, control: -21mins/wk, p=0.03) and MET-hours/week (Int: +17hours/week, control: -8hours/week, p=0.001). |
| Jung, 2011, [5] | Canada | 17- ≤25yrs; 100% female; NR | 133 | I: RD run seminar with targeted gain-framed version. C: RD run seminar | Nutrition | F2F + mail | 14 | University | NA | 0, 1, 8, 25 and 52 weeks | Calcium intake (3-day food record) | Significant diff between grps favouring int grp for calcium intake at 52 wks (+295mg/day, p<0.01) |
| Franko, 2012, [6] | USA | 17- ≤25yrs; 100% female; predominantly non-white | 64 | I: participants completed two computer-based programs adressing eating disorder risk and eating behaviours. C: Accessed two non diet related websites | Nutrition + Eating Disorder risk factors | F2F + online | 2 | University | 3-mon: 80% | 0, 2 weeks, and 3-m | FV intake (self-report short diet question) | Significant between group change in FV intake favouring int group at post test (p<0.001) (Int group: +1.00, control group: +0.20 serves/day) and 3 months (p=0.002) (Int group: +0.77, control group: +0.32 serves/day) |
| Share, 2015, [7] | Australia | 17- ≤30yrs; 100% female; predominantly white | 39 | I: lifestyle program with weekly nutrition and cognitive behaviour therapy session, and twice weekly supervised physical activity sessions. C: Wait list | Nutrition and Physical Activity | F2F | 12 | University | 24-wks: 70% | 0, 12 and 24 weeks | Waist circumference (measured) | No significant between grp difference on waist circumference at 12 or 24 weeks |
| Stice, 2013, Healthy Weight 2, [8] | USA | 17- ≤30yrs; 100% female; predominantly white | 398 | I: weekly one hour group sessions on nutrition and physical activity/prevention of eating disorder symptoms and unhealthy weight gain. C: educational brochure on body image | Nutrition, Physical Activity and Obesity + Eating disorder symptoms | F2F | 4 | University | 24-mon: 93% | 0, 1, 6, 12 and 24-m | BMI (kg/m2) (measured height and weight) | No significant between grp differences in BMI at 6, 12 or 24-mon. |
| Tavakoli, 2016, [9] | Iran | 17- ≤35yrs; 100% male; NR | 280 | I: 2 x 60 minute nutrition education sessions based around the health belief model. C: No education | Nutrition | F2F | NR | Military university | 4-wks: 86% | 0, 4 weeks | Diet behaviour score (self-report questionnaire) | Significant between grp difference in diet behaviour (p<0.05). Int: +0.61, p<0.001). Control: -0.46, p<0.02) |
| Chang, 2010, Mothers in motion, [10] | USA | 17- ≤35yrs; 100% female; predominantly white | 129 | I: 5 DVD presented chapter and 5 peer support teleconferences + 20 minute nutrition education every 6 months (usual care) C: 20 minute nutrition education every 6 months (usual care) | Nutrition, Physical Activity and Obesity + stress management | F2F+ DVD + phone + print materials | 10 | Womens Infant and Childrens clinic | 10.5-mon: 30% | 0, 4.5 and 10.5-m | Weight (lbs) (measured) | No significant difference between groups at any timepoint for weight. |
| Eiben, 2006, Health Hunters, [11] | Sweden | 17- ≤30yrs; 100% female; NR | 40 | I: Customised support package divided into three main themes. PA, Diet, Weight control. Initial face to face counselling and regular personalised contact via email, telephone, group session and booster visits with dietitians. C: waitlist | Nutrition, Physical Activity and Obesity | Phone + email + F2F group session | 52 | Community | 13-mon: 75% | 0, 13-m | Weight (kg) (measured) | Significant between grp difference favouring intervention grp for weight at 1yr (Int: -1.9kg, Con: +2.6kg, p<0.04). |
| Ortega, 2006, [12] | Spain | 17- ≤35yrs; 100% female; NR | 67 | I: Diet C; weight control measures focusing on increasing cereals. I: Diet V; weight control measures focusing on vegetables | Obesity | F2F | 6 | University | 6-wks: 85% | 0, 2 and 6 weeks | Weight (kg), BMI (kg/m2), WC, W:H ratio, skin fold, body composition (measured) | Significant between grp difference favouring diet C for weight at 6-wks (-2.8kg and -2.0kg, p<0.05). No significant between grp difference for other outcomes. |
| Williams, 2002, [13] | USA | 17- ≤25yrs; 100% male; predominantly white | 45 | I: Nutrition counseling, serum cholesterol measurement, or both. 4 x 20-30 minute weekly sessions for those in nutrition counseling groups. C: No intervention. | Nutrition | F2F | 4 | University | 6-wks: 100% | 0, 1 and 6 weeks | %kcal from fat (24-hr recall and 2-day food record) | Significant between group difference for change in %kcal from fat at end of intervention (6-weeks) for Int arm 1 (Counselling + Cholesterol feedback) compared to control (-3.4%, p=0.02). No other significant between group differences. |
| Uglem, 2013, [14] | Norway | 17- ≤30yrs; 100% male; NR | 787 | I: Change in food environment increase in availability of vegetables and semi-wholegrain breads and nutrition information. C: No intervention | Nutrition | F2F + print materials | 20 | Military | 5-mon: 61% | 0, 5-m | Vegetables & semi-whole grain bread (grams/day) (4-day food diary) | Significant between group differences at end of program (5-m) favouring int group for vegetables (+137 grams/day, p<0.001) and semi whole grain bread (+56 grams.day, p<0.001). |
| Hutchesson, 2018, Be positive Be Healthe, [15] | Australia | 17- ≤35yrs; 100% female; predominantly white | 57 | I: BPBH supported participants to modify diet and physical activity behaviours using evidenced-based strategies (e.g., self-monitoring) tailored for young women and delivered using e-health (website, social media, smartphone application, email, text messages). C: Waitlist | Nutrition, Physical Activity and Obesity | SMS, Website, Email, Social media, app | 26 | University, community, technical college, local businesses, social media | 6-mon: 75% | 0, 6-m | Weight (kg) (measured) | No significant between grp difference for weight |
| Jauho, 2015, [16] | Finland | 17- ≤25yrs; 100% male; NR | 276 | I: Participants given wrist worn PA monitor displaying MVPA time, The time spent on different PA levels, steps and calories. C: Blinded to device given only time of day | Physical Activity | F2F + wearable device | 12 | Military | 3-mon: 76% | 0, 3-m | MVPA (accelerometer), sedentary time (self-report questionnaire) | MVPA increased (p = 0.012) and sedentary time decreased (p = 0.032) in the intervention group compared with the control group over time. Average time in MVPA during the trial 63mins/week and 61mins/week for int and control (p=0.012). |
| Katterman, 2016, [17] | USA | 17- ≤25yrs; 100% female; predominantly white | 50 | I: Participants assigned to daily weight monitoring were given a digital scale and asked to weigh themselves each morning when they woke up and record it on a secure website. C: No intervention | Obesity | F2F + online | 8 | University | 20-wks: 94% | 0, 8 and 20 weeks | Weight (kg) (measured) | No significant between grp difference on weight at 8-wk or 20-wk in entire sample. |
| Katterman, 2014, [18] | USA | 17- ≤30yrs; 100% female; predominantly white | 58 | I: 8 group sessions with topics on healthy eating and exercise. Participants were told that the goal was to help them establish healthy eating and exercise behaviours that would promote long-term weight control and that they may choose to focus on weight gain prevention or “healthy weight loss”. C: No Intervention | Nutrition, Physical Activity and Obesity | F2F group sessions | 16 | University | 52-wks: 64% | 0, 6, 16 and 52 weeks | Weight (kg), BMI (kg/m2) (measured) | No significant between group differences at post intervention. Significant differences favouring intervention at 1 yr for weight (p=0.008) (control +1.07 kg, intervention -2.24kg) and BMI (p=0.015) (control +0.34 kg/m2, intervention -0.74 kg/m2) |
| Klem, 2000, [19] | USA | 17- ≤35yrs; 100% female; predominantly white | 102 | I1: Correspondence course subjects received 10 mailed lessons over a 10 week period and were asked to return a brief homework assignment. I2: Weekly group meetings; participated in weekly group meetings over 10 weeks. C: Lifestyle brochure and make recomended changes | Nutrition, Physical Activity and Obesity | Int 1: F2F + Print materials. Int 2: F2F | 10 | Community and University | 6-mon: 56% | 0, 10 weeks and 6-m | Weight (kg) (measured) | I2 lost significantly more weight than control (p<0.05) (-1.9kg vs -0.2kg) at 10 wks, difference between I1 and control not significant. No significant between group differences at 6-mon. |
| Ornes, 2007, [20] | USA | 17- ≤35yrs; 100% female; NR | 121 | I: Intervention group wore pedometers, set goals, recorded steps taken, web-based intervention C: sealed pedometer control group wore sealed pedometers, no feedback on steps taken, no intervention, C: unsealed pedometer control group wore unsealed pedometers, recorded steps taken only, no intervention. | Physical Activity | Online | 4 | University | 4-wks: 93% | 0, 4 weeks | Steps (pedometer) | Sig differences between control and intervention (p=0.001), intervention group increased steps by 38.8% (2994 steps/day). Sealed pedometer group decreased steps by 13.7% (1022 steps/day) and unsealed pedometer increased by 10.4% (1084 steps/day) |
| Rote, 2015, [21] | USA | 17- ≤25yrs; 100% female; predominantly white | 63 | I: Facebook support group to increase physical activity. C: Standard walking intervention | Physical Activity | Social media | 8 | University | 8-wks: 84% | 0, 8 weeks | Steps (pedometer) | Significant between grp difference for steps/day favouring the intervention grp (Facebook) over control (walking grp) at end of intervention (8-wks) (F(8,425) = 3.78, P < .0004). Int group +7177 steps/day, control +4541 steps/day |
| Sriramatr, 2014, [22] | Thailand | 17- ≤25yrs; 100% female; NR | 220 | I: 6 step web based PA intervention & provided pedometer with or without pre testing. C: Provided Pedometer with or without pretesting | Physical Activity | Online + email | 12 | University | 6-mon: 80% | 0, 3 and 6-m | Steps per day (pedometer), weekly leisure time activity score (self-report questionnaire) | Signficant between grp difference at 3-m and 6-m for steps/day (+3776) and leisure time activity score (+16) (all p<0.01), favouring INT grp over control. |
| Tobias, 1977, [23] | USA | 17- ≤30yrs; 100% female; NR | 96 | I1 (weight reduction manual): Told that obesity was result of learned maladaptive eating patterns and could be instilled through application of various techniques. I2 (Self-determination group): obesity resulted from a failure to exert sheer determination or will power- told they would get results if they realised they must assume responsibility- brief weekly reminders. I3 (Behavioral contract): had self appointed material reward held in escrow until completion, additionally signed a legal contract that reward would be surrendered in the event of failure. C1 (Effort control): Wait list but encouraged to lose weight on their own. C2 (No-contract control): wait list | Obesity | Group sessions + mail | 10 | University | 14-wks: 67% | 0, 10 and 14 weeks | Weight (lb) (measured) | Signficant between group difference favouring the manual and contract groups at 10-wks for weight compared to control (p<0.05). Significant between group difference for weight at follow-up for the manual and contract groups (-6.11 and -6.58 pounds) compared with control (-1.61 pounds), p < .01. |
| Butryn, 2011, [24] | USA | 17- ≤35yrs; 100% female; predominantly white | 54 | I: 2 x 2hr group sessions designed to help participants develop willingness skills, become more mindful, defuse from distressing thoughts about exercise, and strengthen their commitment to exercise-related values. C: education intervention provided information about safely engaging in physical activity. | Physical Activity | Group sessions | 5 | University | 8-wks: 85% | 0, 5 and 8 weeks | Gym attendance (electronic swipe card entry data) | Significantly greater increse in gym visits for intervention vs control at 5-wks (+0.75 and -0.17 days/week, p<.05). Difference no longer significant at 8-wks. |
| Valve, 2013, LINDA, [25] | Finland | 17- ≤25yrs; 100% female; NR | 3059 | I: 20 minute individualised lifestyle counselling session followed by further support at the six-monthly follow-up visits of the vaccination trial. C: Counselling on sexual health and contraception | Nutrition and Physical Activity + sleep | F2F + print materials | 78-130 | Health centre | 1.5/2.5yrs: 88% | 0, 1.5/2.5yrs | BMI (kg/m2) (measured height and weight), meal regularity, time exercising and sedentary time (self-report questionnaire) | No signifcant between group differences in BMI (p=0.769), meal regularity for breakfast (p=0.440), lunch (p=0.730) or dinner (p=0.629), time exercising or sedentary time (p=0.771) at end of program. |
| Leinonen, 2017, [26] | Finland | 17- ≤25yrs; 100% male; NR | 496 | I: given a wrist-worn physical activity monitor with feedback, and access to a gamified web-based mobile service, providing fitness guidelines, tailored health information, advice of youth services, social networking, and feedback on physical activity. C: Provided with similar PA monitor which provided no feedback. | Physical Activity | Wearable device | 24 | Military | 6-mon: 71% | 0, 6-m | MVPA time (wrist-worn activity monitor) | No sig between group difference on MVPA |
| Matvienko, 2001, [27] | USA | 17- ≤30yrs; 100% female; predominantly white | 40 | I: College based nutrition science course with lectures and laboratory exercises. C: No course | Nutrition, Physical Activity and Obesity | F2F | 16 | University | 16-mon: 8% | 0, 4 and 16-m | Weight (kg) (measured) | No significant between group difference in weight |
| Bailey, 2019, [28] | USA | 17- ≤25yrs; 100% female; NR | 120 | All groups assigned daily step count goals and sent daily text message to encourage completion. I1: 10,000 steps/day. I2: 12,500 steps/day. I3: 15000 steps/day | Physical Activity | Wearable device + text message | 24 | University | 6-mon: 77% | 0 and 6-mon | Weight (kg) (measured) | No significant between group differences in weight |
| Memon, 2018, [29] | Pakistan | 17- ≤25yrs; 100% female; NR | 56 | I1: incentivized group - self-monitoring of steps with weekly financial incentive based on steps/day, increasing with number of steps recorded. I2: non-incentivized group - self-monitoring with no incentive | Physical Activity | Smartphone app | 5 | University | 5-wks: 100% | 0 and 5-wks | Weight (kg) (measured), steps (measured – smartphone app) | No significant between groups difference in weight or steps |
| Amiot, 2018, [30] | Canada | 17- ≤30yrs; 100% male; NR | 32 | I: F2F individual information session including the negative impacts of eating meat, tips on reducing intake and to set goals for meat intake for the following month. Follow-up text messages daily for 2 weeks about benefits of reducing meat intake. C: No intervention | Nutrition | F2F + eHealth | 2 | University | 2-wks: 100%; 4-wks: 100% | 0, 2 and 4-wks | Meat intake (grams/day) (3-day weighed food record) | No significant between group difference post intervention. Significant reduction in total meat intake in intervention compared with control at follow up (I:-169.68g/day, C: -105.62g/day) and in total red meat intake (I:-186.07g/day, C:-54.82g/day) |

**Table S3:** Detailed study characteristics of included studies - Gender-neutral studies (n=77)

| **First author, year, study, name, citation** | **Country** | **Study participants (age range; % male/female; ethnicity)** | **N at baseline** | **Intervention (I)/Comparator** | **Intervention Focus (Nutrition/Physical activity/ Obesity)** | **Delivery Mode** | **Intervention Duration (weeks)** | **Recruitment setting** | **Retention** | **Data collection timepoints** | **Specific outcome primary measure** | **Effect (between group difference)** |
| --- | --- | --- | --- | --- | --- | --- | --- | --- | --- | --- | --- | --- |
| Bertz, 2015, [31] | USA | 17- ≤25yrs; 54% female; predominantly white | 167 | I: Daily self-weighing and e-mailed feedback (graph of weight change). C: Self-weigh on 3 days/week once every 6 months, no feedback. | Obesity | Video lecture and email | 52 | University | 12-mon: 78% | 0, 6 and 12-m | Weight (kg) (measured) | Significant between group difference in weight at 1 year (control +1.1kg SD: 4.4, Int -0.5kg SD 3.7, p=0.04). |
| Brookie, 2017, Let them eat fruit, [32] | NZ | 17- ≤25yrs; 67% female; predominantly white | 174 | I1: challenged to increase FV to 5 servings/day, received a $10 voucher to purchase FV, + 2 dietary SMS/ day for 13 days. I2: challenged to increase FV by 1 serving/day and received FV package. C: received sugar-free chewing gum and asked to consume one piece/day. | Nutrition | I1: F2F + SMS. I2: F2F | 2 | University | 2-wks: 100% | 0, 2 weeks | Fruit and vegetables (self-report short diet questions) | Significant between grp differences for FV servings in the EMI (M= +3.72, SE=0.15, p< 0.001) and FVI conditions (M=+3.67, SE= 0.15; p< 0.001) compared with control (M=2.81, SE=0.15) at 2-wks. No significant differences between EMI & FVI conditions (p=0.976). |
| Brown, 2014, MobileMyPlate, [33] | USA | 17- ≤25yrs; NR; predominantly white | 150 | I: MMS with 7 behavior-directed motivational Dietary Guideline messages. C: Brochure at the beginning of the intervention containing the MyPlate icon along with the same 7 messages. | Nutrition | SMS | 7 | University | 7-wks: 77% | 0, 7 weeks | Fruit, Vegetables (self-report FFQ) | Significant between group difference favouring the INT group for fruit (p<0.05), but not vegetables at 7-wks. Actual intakes not reported. |
| Buscemi, 2011, [34] | USA | 17- ≤35yrs; 14% female; predominantly non-white | 70 | I: 1x50–60-min information session encouraging increase PA, monitor portion size, increase FV and decrease fast food and SSB's; handout with tips to change diet and exercise; booster phone call at 2-wks. C: Provided information about on-campus recreation centre and web sites for diet/exercise information | Obesity, Nutrition and Physical Activity | F2F + eHealth + Print materials | 12 | University | 3-mon: 86% | 0, 3-m | BMI (kg/m2) (measured height and weight) | No significant between group difference for BMI at 3-mon (β=0.11, p=0.64). |
| Chapman, 2009, [35] | UK | 17- ≤25yrs; 74% female; predominantly white | 557 | I1: Message encouraging 5 FV serves/day, followed with instructions to write plans to increase intake using an if, then format. I: identical intervention without format of writing plan. C: No intervention | Nutrition | F2F | Single session | University | 1-wk: 54% | 0, 1 week | Fruit and vegetables (self-report short diet question) | Significant between grp difference in FV serves/day at 1-wk (p<0.01). Within-grp differences: If-then group +0.5 serves/day, p<0.01; Global group +0.31 serves/day, p=0.01; Control group +0.01 serves/day, p=0.89. |
| Hivert, 2007, [36] | Canada | 17- ≤30yrs, 81% female; predominantly white | 115 | I: Small group seminars designed by an endocrinologist, dietitian and physical education specialist. C: no intervention | Obesity, Nutrition and Physical Activity | F2F | 104 | University | 24-mon: 83% | 0, 3, 6, 12, 18 and 24-m | BMI (kg/m2), Weight (measured) | Significant between group differences at 2 years favouring intervention group for changes in weight (-1.3kg, p=0.04) and BMI (-0.5kg/m2, p=0.01). |
| Werch, 2010, project fitness, [37] | USA | 17- ≤25yrs; 59% female; predominantly white | 283 | I: one-on-one 25 minute consultation using a protocol with scripted messages. Participants received a one-page goal plan. C: Generic information brochue on fitness | Physical Activity and Nutrition + sleep, stress, alcohol, smoking | F2F | Single session | University and Health service | 12-mon: 77% | 0, 3 and 12-m | FV, CHO, fats (self-report short diet questions), moderate exercise, vigorous exercise (self-report questionnaire) | Significant interaction for past 30 days moderate exercise favouring intervention group at 3 months (+0.46 score) and 12 months (+0.52 score), p=0.04. No significant between grp diffs on diet outcomes at 3-m (not analysed at 12-mon) |
| Zhang, 2012, [38] | UK | 17- ≤25yrs; NR; NR | 173 | I1: Motivational Intervention: received leaflet, designed to target PMT variables in relation to type 2 diabetes, I2: Volitional intervention: completed action and coping planning sheets. I3: Combined intervention (I1&I2). C: No intervention | Nutrition and Physical activity | F2F + Print materials | Single session | University | 4-wks: 48% | 0, 2 and 4 weeks | Energy from fat, FV intake (self-report FFQ), Days of exercise (self-report questionnaire) | Combined intervention significantly reduced % energy from fat (-4.82%) compared with motivational, volitional and control (+0.38%, +0.37% and +0.7%), p<0.01, at 4 wks. Motivational and combined intervention significantly increased FV intake (+0.18 and +0.77 serves/day) compared with volitional and control groups (+0.11 and no change in serves/day) p=0.02. Volitional and combined intervention significantly increased PA (+0.23 and +0.49 days/week) compared with motivational and control (-0.01 and +0.01 days/week) p<0.01. |
| LaChausse, 2012, MyStudentBody, [39] | USA | 17- ≤35yrs; 76% female; predominantly non-white | 358 | Courses providing nutrition and physical fitness education for 2hrs/week delivered either online (I1) or F2F on-campus (I2). C: No intervention | Obesity, Nutrition and Physical Activity | I1: Online + print materials I2: In person + print materials | 12 | University | 14-wks: 87% | 0, 14 weeks | Fruit intake, vegetable intake (self-report short diet questions), frequency of aerobic exercise (self-report questionnaire), BMI (self-report height and weight) | Significant between group differences for I1 vs I2 and control for fruit (+0.7, +0.01 and -0.09 serves/day, p=0.003) and for veg intake (+0.36, +0.14 and +0.15 serves/day, p=0.04) at 14-wks. No significant between group differences in BMI or aerobic exercise. |
| LaRose, 2010, [40] | USA | 17- ≤35yrs; 98% female; predominantly white | 52 | I1: Self regulation with large changes. I2: Self regulation with small changes. Both groups attended 8 weekly, then 2 monthly meetings. | Obesity, Nutrition and Physical Activity | F2F | 16 | Community | 4-mon: 84.6% | 0, 2 and 4-m | Weight (kg) (measured) | Significant between group differences at 4-mon: large changes group -3.5kg, small changes group -1.5kg, p=0.006. |
| Laska 2016, CHOICES, [41] | USA | 17- ≤35yrs; 68% female; predominantly white | 441 | I: Academic course and social network support website. C: Health assessments | Obesity, Nutrition and Physical Activity | F2F and online | 104 | University | 24-mon: 93% | 0, 4, 12 and 24-m | BMI (kg/m2) (measured height and weight) | No significant bteween group difference in BMI at 24-mon. |
| LeCheminant, 2011, [42] | USA | 17- ≤25yrs; 63% female; predominantly white | 47 | I: Provided pedometer, goal of 10,000 steps/ day, a handout with suggestions to achieve this goal, an activity record to be completed weekly, and sent weekly reminders to record/information to support activity. C: Advised to continue current PA. | Physical Activity | F2F, print materials and online | 28 | University | 7-mon: 67% | 0, 7-m | PA (self-report questionnaire), VO2 max (measured – treadmill test), weight, WC & body fat % (measured) | No significant between group differences in self reported PA, VO2max, weight, waist circumference, or body fat percentage at 7-mon (p>0.05). |
| Lhakhang, 2014, [43] | India | 17- ≤30yrs; 52% female; NR | 224 | I1: Sequence 1 group received a motivational information package after the baseline measurement (T1) and a self-regulatory information package after the post-test (T2) I2: Sequence 2 group received the information packages in the opposite order | Nutrition | F2F + Print materials | 2.4 | University | 34-days: 92% | 0, 17 and 34 days | Fruit and vegetables (self-report short diet questions) | Sequence 1 group reported higher FV intake levels (M = 6.97, SE = 0.20) than those of the Sequence 2 group at 34-days (M = 5.89, SE = 0.19), F(1, 194) = 15.72, p < .001. Change, sequence 1: +2.69 portions/day, sequence 2: +0.61 portions/day |
| Lua, 2013, [44] | Malaysia | 17- ≤25yrs; 88% female; NR | 417 | I: Nutrition education delivered via three modes; conventional lecture, three brochures as take home messages and text messages as reinforcement. C: No intervention | Nutrition | F2F, SMS + Print materials | 10 | University | 10-wks: 91% | 0, 10 weeks | Weight (kg) (measured), MET min per week (calculated from self-report questionnaire) | Total MET mins/week significantly increased in intervention vs control (mean diff 1545.8 (95% CI 1129.4, 1962.2), p<0.001). No significant difference in weight (p>0.05). |
| Maher, 2015, [45] | USA | 17- ≤30yrs; 50% female; predominantly white | 195 | I: 2×2 factorial design; action plan describing when, where, and how they would engage in physical activity the following day (Factor 1), or when, where, and how they would limit or interrupt an extended period sitting the following day (Factor 2). | Physical Activity | F2F + email | 1 | University | 1-wk: 96% | 0, 1, 2, 3, 4, 5, 6, 7 days | MET min per day (calculated from self-report questionnaire) | No significant between group differences in PA at 1-wk. |
| Martens, 2012, [46] | USA | 17- ≤30yrs; 82% female; predominantly non-white | 70 | I: single session 30 minute 1-on-1 brief motivational intervention. C: Educational brochure only | Physical Activity | F2F | Single session | University | 1-mon: 98% | 0, 1-m | Mod PA (mins/week and days of 30+ mins/ week), vig PA (mins/week and days of 20+ mins/ week) (self-report questionnaire) | Intervention group significantly increased vig PA compared with control for days of 20+ mins/week (+1.3 days, +0.6 days, p=0.02) and mins/week (+45 mins, +10 mins, p=0.01) at 1-mon. No significant differences for mod PA. |
| Meng, 2017, [47] | USA | 17- ≤25yrs; 67% female; predominantly white | 338 | I: self-tracking of fruit and vegetable intake three times per week sub categorised into four interventions (1) similar demograhic x incremiental change, (2) similar demograhic x ideal change, (3) diverse demographic x incrimental change, (4) diverse demographic x ideal change. C: Reported fruit and vegetable intake without presence of group members. | Nutrition | Online | 4 | University | 4-wks: 23% | 0, 4 weeks | Fruit and vegetable intake (self-report FFQ) | Intervention groups consumed more FV than control group (p=0.01) at 4-wks. Diff: +1.12-1.45 vs -0.57 serves/day |
| Napolitano, 2013, [48] | USA | 17- ≤30yrs; 86% female; predominantly white | 52 | I1: Facebook or I2: Facebook and text message delivered weight loss program. C: Wait list control | Obesity, Nutrition and Physical Activity + stress | I1: Online, I2: Online and SMS | 8 | University | 8-wks: 96% | 0, 4 and 8 weeks | Weight (kg) (measured) | Facebook Plus group had significantly greater weight loss (-2.4kg) than Facebook group (-0.63kg) and control (-0.24kg), p<0.05. |
| Nix, 2017, [49] | USA | 17- ≤25yrs; 76% female; predominantly white | 167 | I: Email with one of 3 message themes; recommendation (number of fruit and vegetables recommended), high norm (other students eat less FandV than you), low-norm (other students eat more FandV than you). C: No feedback | Nutrition | Email | Single session | University | 1-wk: 100% | 0, 1 week | FV intake (self-report short FFQ) | Significant mean difference in FV intake between low and high norm groups favouring low norm group (+0.6 vs -0.1cups/day, p=0.035). No other between group differences. |
| O'brien, 2016, [50] | USA | 17- ≤30yrs; 68% female; predominantly white | 154 | I1: web based intervention with personalised feedback I2: web-based intervention with personalised feedback and daily text messages C: Online assessment only. | Nutrition and Physical Activity | I1: Online I2: Online and text messages | 4 | University | 1-mon: 96% | 0, 1-m | Fruit and vegetable intakes (self-report short diet questions) | Significant difference in achieving veg guidelines for I2 compared to control (adjusted odds ratio= 2.93, 95% CI: 1.06, 8.12, p = 0.04). No significant difference in fruit intake |
| Park, 2008, [51] | USA | 17- ≤25yrs; 67% female; predominantly white | 111 | I: Transtheoretical model treatment intervention. C: Standard action orientated messages based on standard 5-A-day messgae | Nutrition | Online | Single session | University | 60-days: 87% | 0, 30-60 days | Fruit and vegetable consumption (self-report short diet questions) | No significant between group difference in fruit or vegetable consumption |
| Pearson, 2013, CHANGE program, [52] | Canada | 17- ≤25yrs; 76% female; NR | 45 | I1: unscripted motivational interview telephone intervention I2: Scripted education based intervention | Obesity, Nutrition and Physical Activity | Telephone | 12 | University | 12-mon: 58% | 0, 6, 12 weeks, and 6, 12-m | Weight (lb) (measured) | I2 Participants decreased weight more than I1 at 12-wks (Mean -7.76lb (SE 2.05) vs -2.5 (1.70), p=0.05. Effect size: 0.29. No other significant differences at 3 or 6 months. |
| Kypri, 2005, [53] | New Zealand | 17- ≤25yrs; 49% female; predominantly white | 218 | Computerised assessment and advice on fruit and vegetable consumption, PA, alcohol and smoking with (I1) and without feedback (I2) C: No intervention | Physical Activity and Nutrition + alcohol and smoking | Online | Single session | University | 6-wks: 83% | 0, 6 weeks | FV intake (self-report short diet questions) and PA (self-report questionnaire) (meeting guidelines) | Significant between group differences for I1 vs control for meeting FV recommendations (33% vs 13%, p=0.02) and PA recommendations (90% vs 71%) at follow up. No other between group differences. |
| Gokee-LaRose, 2009, [54] | USA | 17- ≤35yrs; 87% female; predominantly white | 40 | I1: behavioural self regulation education- given scales and education on principles of self regulation. I2: non-weigh group with emphasis on behaviour not weight. Both groups received intervention over 10x weekly 60 min sessions + booster session at week 14 | Obesity, Nutrition and Physical Activity | F2F | 14 | Community | 20-wks: 85% | 0, 10 and 20 weeks | Weight (kg) (measured) | No significant between group difference in weight at post-treatment [BSR = -6.4 kg (4.0); SBT = -6.2 kg (4.5)], F (2, 76) = .170, p = .84, or 20 week follow up [BSR = -6.6 kg (5.5); SBT = -5.8 kg (5.2)], F (2, 76) = .170, p = .84. |
| Gow, 2010, [55] | USA | 17- ≤25yrs; 74% female; predominantly white | 159 | I1: Weekly weigh in with online reporting/feedback. I2: Weekly online intervention sessions adressing healthy eating, PA, media literacy and positive body image. I3: Combined group i.e. I1 and I2. C: No intervention | I1: Obesity I2 & I3: Obesity, Nutrition and Physical Activity | Online | 6 | University | 3-mon: 11% | 0, 6 weeks, and 3-m | BMI (kg/m2) (measured height and weight) | Significant between grp difference with combined group (feedback and online) had lower BMI at 6-wks comapred to other 3 groups. The combined intervention group (M = 24.13,SE = .09) had significantly lower BMI scores than the control group (M = 24.56,SE = .09,p <.05). Not analysed/reported for 3-mon due to high attrition rate. |
| Hebden, 2014, [56] | Australia | 17- ≤35yrs; 80% female; NR | 51 | I: mHealth adressing PA, sedentary behaviour, fruit and vegetables, energy dense take away food, and SSB via SMS and email + single session with dietitian/booklet. C: single session with dietitian + booklet covering core food groups, meal plan and exercise | Obesity, Nutrition and Physical Activity | SMS, email, online forum, F2F + print materials | 12 | University | 12-wks: 90% | 0, 12 weeks | BMI (kg/m2),  weight (kg) (measured height and weight) | No sig between grp difference in weight or BMI at 12-wk. |
| Kerr, 2016, CHAT, [57] | Australia | 17- ≤30yrs; 66% female; predominantly white | 247 | I: Dietary feedback and weekly text messages or Dietary feedback alone. C: No intervention | Nutrition | Text messages | 24 | Community | 6-mon: 89% | 0, 6-m | Fruit, Veg, SSB, EDNP serves/day (self-report questionnaire) | No signficant between grp differences on any diet outcome at 6-month. |
| Allman-Farinelli, 2016, TXT2BFIT, [58] | Australia | 17- ≤35yrs; 61%; NR | 250 | I: Booklet with meal plans, recommended food group servings, and information about four target behaviours; PA and sedentary behaviour, fruit and vegetable intake, energy dense take-away foods and SSB in conjunction with TXT2BFit mobile phone based program (SMS, email, app, coaching calls and website). C: Booklet, introductory call without coaching, four SMS messages and limited access to website | Obesity, Nutrition and Physical Activity | Phone based (SMS, email, app and website) and print materials | 36 | Community and university | 9-mon: 81% | 0, 3 and 9-m | Weight (kg) (measured) | Significant difference in weight change between groups at 9-months: 4.3 kg (95% CI −6.9 to −1.8, P=0.001) |
| Svetkey, 2015, CITY, [59] | USA | 17- ≤35yrs; 70% female; predominantly white | 365 | I1: Information, self-monitoring and feedback via smartphone app. I2: Information and feedback via an interventionist (F2F and over the phone), supported by smartphone app for self-monitoring. C: Information handouts on nutrition and physical activity | Obesity, Nutrition and Physical Activity | I1: App. I2: F2F, phone calls and app | 104 | Community and university | 24-mon: 86% | 0, 6, 12 and 24-m | Weight (kg) (measured) | I2 had significantly greater weight loss (−3.07, −3.58, and −2.45 kg at 6, 12, and 24 months, respectively), than control (−1.92 kg [CI −3.17 to −0.67], p=0.003), and I1 at 6 months (−2.19 kg (CI −3.42 to −0.97], p<0.001) and I1 at 12 months (−2.10 kg [CI −3.94 to −0.27], p=0.025). No significant between group differences in weight loss at 24 months (primary outcome). |
| Kattelmann, 2014, YEAH, [60] | USA | 17- ≤25yrs; 67% female; predominantly white | 1639 | I: Weekly F2F educational lessons reinforced with email messages (nudges). C: Waitlist control | Obesity, Nutrition and Physical Activity + stress | F2F and email | 10 | University | 15-mon: 59% | 0, 10 weeks, and 15-m | Weight (kg), BMI (kg/m^2^) (measured height and weight), FV intake (self-report short FFQ), PA (self-report questionnaire) | No significant differences between groups at any timepoint for all weight outcomes or PA. Significant difference with a small effect size between experimental and control at postintervention for FV itnake (diff +0.4cups/day), which was not sustainted at 15-mon. |
| Do, 2008, [61] | USA | 17- ≤25yrs; 61% female; predominantly white | 2024 | I: Mailed intervention materials tailored to stage of change and two educational phone calls. C: Mailed non-tailored pamphlet | Nutrition | Phone and print materials | 24 | Community | 12-mon: 62% | 0, 4 and 12-m | Fruit, vegetables (self-report FFQ) | Experimental group increased intakes more than control for fruit and vegetable at 4 & 12 months. Change/baseline intake NR |
| Franko, 2008, MyStudentBody, [62] | USA | 17- ≤25yrs; 57% female; predominantly white | 606 | I (arm 1): Instructed to use online nutrition & PA program for two web sessions. I (arm 2): Instructed to use online nutrition & PA program for two web sessions and a subsequent booster session. C (arm 3): Instructed to use an interactive anatomy education website for two web sessions. | Nutrition and Physical Activity | I1: Online I2: Online | 2 | University | 6-mon: 70% | 0, 2 weeks, 3 and 6-m | FV intake (self-report short diet question and FFQ), percent energy from fat (self-report FFQ) | Intervention arm 1 and 2 significantly increased FV at post test compared with control (+0.33 and 0.24 servings, respectively, p<0.01). Fat intake not measured at post test. No difference at 3 or 6mo for FV or fat intake. |
| Goodman, 2016, [63] | Canada | 17- ≤25yrs; 58% female; predominantly white | 109 | I: Behavioural intervention consisting of an educational video, online information and phone app to track vitamin D intake. C: Wait list | Nutrition | Video, online and app | 12 | Community | 3-mon: 83% | 0, 2 weeks and 3-m | Vitamin D (self-report questionnaire) | Mean vitamin D intake significantly increased in intervention vs control (+308 IU vs. 131 IU, respectively). Significantly different from baseline for intervention group (p<0.001) not for control (p<0.05) |
| Heeren, 2017, [64] | South Africa | 17- ≤25yrs; 53% female; NR | 176 | I: Group session health promotion intervention targeting PA, diet and alcohol. C: Attention matched control focused on HIV risk | Physical Activity and Nutrition + alcohol | F2F | 8 | University | 12-mon: 97% | 0, 6 and 12-m | Met PA guidelines (self-report questionnaire) | Intervention group significantly more likely to meet PA guidelines than control (OR=3.35; 95% CI: 1.33–8.41). |
| Kendzierski, 2015, [65] | USA | 17- ≤30yrs; 75% female; predominantly white | 142 | I: Implementation intention condition participants listed what fruits and vegetables they would eat and when and where they would eat them. C: No intervention | Nutrition | F2F + Print materials | Single session | University | 1-wk: 82% | 0, 1 week | Fruit, vegetables (self-report 4-day recall) | Healthy eater schematics in the intervention group consumed more servings of vegetables than control condition (Mean = 3.11 vs 2.18, F(1, 29) = 5.89, p = .022). No significant findings for fruit intake. |
| Kendzierski, 2015, [65] | USA | 17- ≤30yrs; 67% female; predominantly white | 79 | I: Implementation intention condition participants listed what vegetables they would eat and when and where they would eat them. C: No intervention | Nutrition | F2F + Print materials | Single session | University | 1-wk: 95% | 0, 1 week | Vegetables (self-report 4-day recall) | Healthy eater schematics in the intervention group consumed more servings of vegetables than control condition (Mean = 3.41 vs 2.33, F(1, 18) = 4.48, p = .049). |
| Knauper, 2011, [66] | USA | 17- ≤25yrs; 62% female; predominantly white | 247 | I1: Goal intention + Implementation intentions. I2: Goal intention + mental imagery. I3: Goal intention + Mental imagery targeted to the implementation intentions. C: Goal intention only | Nutrition | Online | Single session | University | 1-wk: 96% | 0, 1 week | Fruit intake (self-report short diet question) | Significant overall effect for time x condition for fruit intake (p<0.05). Post hoc, effects only significant for low fruit consumers: I3 > control, I1 & I2. I1 & I2 > control. Change from baseline for low consumers: I1 +1.1, I2 +1.37, I3 +2.06 and control +0.87 portions/day. |
| Kothe, 2014, Fresh Facts, [67] | Australia | 17- ≤25yrs; 83% female; predominantly white | 162 | I: Automated email messages promoting fruit and vegetable consumption every 3 days. Messages targeted attitude, subjective norm and perceived behavioural control. C: baseline and follow up questionnaire | Nutrition | Email | 4 | University | 1-mon: 82% | 0, 1-m | Fruit and vegetable intake (self-report short diet questions) | No significant between group differences for FV intake at 1-mon (p=0.499). |
| Kreausukon, 2012, [68] | Thailand | 17- ≤25yrs; NR; NR | 121 | I: Received a training program including general health and nutrition education plus psychological program focusing on self-efficacy enhancement and planning skills. C: Active control group received only the general health and nutrition education | Nutrition | F2F | Single session | University | 6-wks: 94% | 0, 1 and 6 weeks | Fruit and vegetable intake (self-report short diet questions) | Significant between group difference in favour of intervention for FV intake at 6-weeks (I: +1.29 serves/day, C: +0.8serves/day, p values <0.05). |
| Richards, 2006, [69] | USA | 17- ≤25yrs; 71% female; predominantly white | 437 | I: Four stage based newsletter, one motivational interview, and an individually tailored e-mail follow-up over a 4-month period. C: No intervention | Nutrition | Newsletter, email and F2F | 16 | University | 4-mon: 72% | 0, 4-m | Fruit and vegetable intake (self-report FFQ) | Significant between group difference favouring intervention for FV intake (p<0.001). I: +1.0serves/day, C: +0.4serves/day. |
| Rompotis, 2014, [70] | Australia | 17- ≤35yrs; 82% female; NR | 161 | I1: SMS messages consisting of habit framework principles. Active C1: SMS message active control fruit and vegetable consumption. Active C2: SMS active control general healthy eating. I2: E-mail messages consisting of habit framework principles. Active C3: E-mail message active control fruit and vegetable consumption. Active C4: E-mail active control general healthy eating | Nutrition | I1, AC1-2: SMS I4, AC3-4: Email | 8 | University | 8-wks: 45% | 0, 8 weeks | Fruit, vegetables (self-report short diet questions) | Significant between group difference favouring the habit messages over controls for fruit (p=0.008) but not vegetable intake (p=0.12). Actual intake values not reported. |
| Sandrick, 2017, [71] | USA | 17- ≤30yrs; 68% female; predominantly white | 60 | I: F2F health coaching session focused on results of baseline health behaviour questionnaire + text message support to encourage goal completion. C: Received results from baseline health behaviour questionnaire only. | Physical activity and Nutrition + stress and sleep | F2F + SMS | 8 | University | 8-wks: 97% | 0, 8 weeks | Diet score (self-report questionnaire), METs/week (calculated from self-report questionnaire) | No significant between group difference in diet score (p=0.81). Significant between group difference in MET mins/week favouring the INT group (+646 MET mins/week, p=0.04). |
| Schweitzer, 2016, ALIVE, [72] | USA | 17- ≤25yrs; 69% female; predominantly white | 148 | I: Email delivered tailored and interactive diet and PA goals. C: Fact sheets on alternative health behaviours | Nutrition and Physical activity | Email | 24 | University | 6-mon: 72% | 0, 3 and 6-m | Fat, FV, sugar, and snacks intake (self-report FFQ), MVPA mins/week (calculated from self-report questionnaire) | Significant between group difference favouring intervention in % energy from saturated fat (-1.3%, p=0.048) at 3-mon. No significant between grp differences for any other diet variable or MVPA at 3 or 6-mon. |
| Stephens, 2017, [73] | USA | 17- ≤25yrs; 71% female; predominantly non-white | 62 | I: smart phone application and health coach intervention and counselling session. C: counselling session | Obesity, nutrition and physical activity | F2F, app + SMS | 12 | University | 3-mon: 95% | 0, 3-m | Weight, BMI, WC (measured) | Significant between group difference for weight (-2.1kg, p=0.026), WC (-2.5cm, p<0.01) and BMI (-1.2kg/m2, p=0.024) at 3-mon compared to controls. |
| Annesi, 2015, [74] | USA | 17- ≤30yrs; 66% female; predominantly white | 98 | I: Small and large classes follow the "coach approach" The components of The Coach Approach, include goal setting and progress feedback, cognitive restructuring/positive self-talk, and relapse prevention training, are intended to increase selfregulatory skill usage related to exercising, increasing exercise self-efficacy, and improving mood. C: Usual class process which covered components of fitness, stress, weight management, CVD and nutrition. | Obesity, Nutrition and Physical activity + stress | F2F | 15 | University | 15-wks: 95% | 0, 15 weeks | Days/week exercising (self-report questionnaire) | No significant between group difference on days/wk exercising. |
| Greene, 2012, Project Webhealth, [75] | USA | 17- ≤25yrs; 62% female; predominantly white | 1689 | I: Web based intervention, primarily nutrition and PA focused, weekly 15 min lessons. C: Waitlist | Obesity, nutrition and physical activity | Online | 10 | University | 15-mon: 67% | 0, 3 and 15-m | BMI (kg/m^2^) (calculated from self-report height and weight), Fruit and vegetable intake (self-report short diet questions), MET min per week (calculated from self-report questionnaire) | No significant between group difference in BMI at 15-mon. Significant between group difference favouring intervention group for F&V (+0.5cups/day, p<0.001) and PA (+270 MET-min/wk) (p<0.05) at 15-mon. |
| Ohtsuki, 2018, [76] | Japan | 17- ≤25yrs; 76% female; predominantly non-white | 104 | I: three educational projects; a lesson on the nutritional significance of vegetable consumption, a tour of an agricultural farm, and learning of cooking skills for vegetable intake. Encourage to eat 350 g Veg a day C: habitual lifestyle | Nutrition | F2F | 25 | University | 25-wks: 94% | 0, 25 weeks | Vegetable intake (self-report diet history questionnaire) | No significant between group difference in vegetable intake at 25-wks. |
| Kothe, 2012, Fresh Facts, [77] | Australia | 17- ≤25yrs; 76% female; predominantly white | 194 | I: Automated emails; High frequecy; 27 emails each containing one intervention message targeting increased FV intake. C: active control; 9 longer emails each containing 3 messages | Nutrition | Email | 4 | University | 4-wks: 86% | 0, 4 weeks | Fruit and vegetable intake (self-report short diet questions) | No significant between group difference in FV intake (serves/day) at 1-m. |
| Godino, 2016, Project SMART, [78] | USA | 17- ≤35yrs; 70% female; predominantly white | 404 | I: theory-based, weight loss intervention delivered via integrated user experiences with Facebook, mobile apps, text messaging, emails, a website, and technology-mediated communication with a health coach. Participants were instructed to use at least one modality a minimum of five times per week. C: alternative website focused on other health behaviours and with general weight loss information. Instructed to use this at least weekly. | Obesity, nutrition and physical activity | Facebook, apps, text messaging, email, website, blog posts and online communication | 104 | University | 24-mon: 84% | 0, 6, 12, 18, 24-m | Weight at 24 months (measured) | No significant between group difference in weight at 24 months (–0·79 kg [95% CI –2·02 to 0·43], p=0·204). |
| Pope, 2014, Burn and Earn, [79] | USA | 17- ≤25yrs; 73% female; NR | 117 | I1: continued incentive - received ongoing monetary incentives when meeting fitness centre attendance goals during fall and spring. I2: Discontinued incentive - same incentive as I1 during fall but not continued during spring. C: No incentive | Physical activity | Email | 24 | University | 24-wks: 94% | 0, 12 and 24 weeks | Gym attendance (electronic swipe card entry data) | I1 and I2 significantly higher gym attendance goals met than control in semester 1 (62% and 64% vs 3%, p < .001). I1 significantly higher gym attendance goals met than I2 and control in semester 2 (39% vs 3% and 3%, p<0.001). |
| Walsh, 2016, [80] | Ireland | 17- ≤30yrs; 40% female; NR | 58 | I: App used to record daily steps in conjunction with app providing daily goal number of steps. C: Also used app, not given the feedback. | Physical activity | App | 5 | University | 5-wks: 95% | 0, 5 weeks | Steps per day (pedometer smartphone app) | Significantly greater increase in steps/day in int vs control (2393 vs 1101, p=0.043). |
| Conner, 2011, EXP 2, [81] | UK | 17- ≤30yrs; 72% female; NR | 121 | I1: Information message about the affective benefits of exercising + images. I2: Information message about the instrumental benefits of exercising + images. C: No intervention. | Physical activity | Print materials | Single session | NR | 3-wks: 92% | 0, 3 weeks | Time in exercise per week (self-report questionnaire) | Significantly higher exercise in affective group than cognitive and control (p<0.05), actual data not reported. |
| Cooke, 2013, [82] | UK | 17- ≤25yrs; 60% female; predominantly white | 136 | I: 10 min group talk on health benefits of walking + strategies to increase, set step goal for upcoming week and recorded. C: Recorded daily steps only | Physical activity | F2F | 1 | University | 1-wk: 88% | 0, 1 week | Steps per day (pedometer) | Significantly greater increase in steps/day in intervention group than control (+1310 steps/day, p=0.0001) |
| Cooke, 2014, [83] | UK | 17- ≤25yrs; 50% female; NR | 80 | I1: Self-affirmation activity + read factsheet on health benefits of PA. I2: Non-affirmation activity + read factsheet on health benefits of PA | Physical activity | F2F + Print materials | Single session | University | NR | 0, 1 week | Time in exercise per week (self-report questionnaire) | I1 participants engaged in significantly more activity (M = 77.90, SD = 31.52) than I2 (M = 44.48, SD = 31.45; p<.001). |
| Eisenberg, 2017, [84] | USA | 17- ≤25yrs; 36% female; predominantly white | 159 | I1: E-diary, I2: Accelerometer, or I3: E-diary & accelerometer to measure PA. C: No intervention. | Physical activity | I1: Online. I2: Wearable device. I3: Online + wearable device | 1 | University | 1-wk: 92% | 0, 1 week | METs (calculated from self-report questionnaire), steps (accelerometer), perceived PA (self-report questionnaire) | No significant between group differences for METs or accelerometer steps/day. Perceived PA (METs) was siginificantly higher in acceleromter only group compared to other groups (absolute mean difference 1474-1756 MET minutes/week, p<0.05). |
| Jakicic, 2016, IDEA, [85] | USA | 17- ≤35yrs; 71% female; predominantly white | 470 | I: Technology enhanced group: provided wearable activity tracker with web-based interface for feedback + standard weight loss intervention. C: Standard weight loss intervention: theory based strategies provided during weekly group sessions in the first 6 months, then monthly group sessions and phone calls, text messages and a website for the next 18 months. | Obesity, nutrition and physical activity | I: F2F, phone calls, text messages, website + wearable device. C: F2F, phone calls, text messages + website | 104 | University | 24-mon: 75% | 0, 6, 12, 18, 24-m | Weight (kg) (measured) | Significant difference in weight change favouring Int at 24-mon, mean weight change -3.3kg (95% CI: 2.5 to 4) vs -5.3kg (4.5-6.2), p=0.003. |
| Phimarn, 2017, [86] | Thailand | 17- ≤25yrs; 65% female; NR | 112 | Counselling sessions (x3 over 6 months) on nutrition and exercise, plus a weight loss handbook, in either group sessions (I1) or individual sessions (I2). | Obesity, nutrition and physical activity | F2F + Print materials | 24 | University | 6-mon: 96% | 0, 3 and 6-m | Weight (kg) (measured) | Significant between group difference at 6-mon favouring I2 over I1 for weight (-1kg, p= 0.04). |
| Sharp, 2016, [87] | Canada | 17- ≤25yrs; 53% female; predominantly white | 184 | I: Participants provided with a pedometer, monthly tracking logs and follow up educational/ reminder emails. C: No intervention | Physical activity | Email, wearable device and print materials | 12 | University | 12-wks: 75% | 0, 12 weeks | PA mins/week (self-report questionnaire) | No significant difference between groups at 12-wks for overall PA mins/week (p=0.28). |
| Strohacker, 2015, [88] | USA | 17- ≤25yrs; 69% female; predominantly non-white | 22 | I: Incentive $0.01 for every 4 kilocalories expended through moderate-intensity treadmill or cycling exercise at on campus gym. C: No incentive | Physical activity | F2F | 10 | University | NR | 0, 10 weeks | Caloric expenditure (electronic data capture on exercise machines) | No significant between grp difference on caloric expenditure at 10-wks. |
| Bray, 2011, [89] | Canada | 17- ≤25yrs; 61% female; predominantly white | 935 | I1: Physical activity and action-planning brochure. I2: Canada’s Physical Activity Guide brochure. C: No intervention | Physical activity | Print materials | Single session | University | 6-wks: 27% | 0, 6 weeks | Time in MVPA (self-report questionnaire) | Significant difference between I1 and C at 6-wks (347.8 vs 269.8 mins/week, p=0.03). |
| Calfas, 2000, Project GRAD, [90] | USA | 17- ≤30yrs; 54% female; predominantly white | 338 | I: Course designed to promote PA, including educational and self-monitoring components. C: Lecture covering general health. | Physical activity | F2F, phone and print materials | 78 | University | 24-mon: 93% | 0, 12 and 24-m | TEE, time in MVPA (self-report questionnaire) | No significant between group differences on TEE, time in vigorous PA or time in moderate PA at 2 yrs for either men or women. |
| Wing, 2016, SNAP, [91] | USA | 17- ≤35yrs; 78% female; predominantly white | 609 | 10x F2F meetings over 4 months, followed by online refresher courses, and received quarterly newsletter and feedback reports. I1: Instructed to make small changes (approximately 100 kcal/d) in diet and PA, provided pedometers and a goal to add 2000 steps per day above baseline, and to adjust in case of weight gain. I2: Instructed to make large changes with focus on weight loss. Prescribed a calorie goal (500-1000kcal deficit) for first 8 weeks, gradually increase mod PA to 250 minutes/week. C: Self-guided condition receive one face-to-face group session on health behaviours, weight gain and health consequences. | Obesity | I1: F2F, online and wearable device. I2: F2F and online | 156 | community | 48-mon: 72% | 0, 4, 12, 24, 36 and 48-m | Weight (kg) (measured) | Significantly less weight gain in I1 (P= .02) and I2 (P< .001) vs C, and in I2 relative to I1 (P< .001). Mean (SE) weight change was 0.26 (0.22),−0.56 (0.22), and −2.37 (0.22) kg in C, I1 and I2 groups respectively. |
| Weinstock, 2014, [92] | USA | 17- ≤25yrs; 64% female; predominantly white | 31 | I1: 1x50 minute motivational enhancement therapy (MET) session focused on increasing PA + 8 weeks of contingency management. I2: 1x50min MET session only. | Physical activity | F2F | I1: 8. I2: Single session | University | 2-mon: 94% | 0, 2-m | Frequency, weekly minutes and weekly calories expended from exercise (accelerometer/ self-report questionnaire), and VO2 peak (ergometer test) | Significant between group difference favouring I1 for exercise frequency (+1.5 vs +0.4 days/week, p=0.001). All other PA outcomes were not significant. |
| Weinstock, 2016, [93] | USA | 17- ≤25yrs; 56% female; predominantly white | 70 | I: 2x 50min motivational interviewing sessions (MI) + weekly exercise contracting. C: 2x 50 min MI sessions + weekly contingency management for exercise. | Physical activity | F2F | 8 | University | 6-mon: 86% | 0, 2 and 6-m | Exercise frequency, METs hrs/week (self-report questionnaire), VO2 max (ergometer test) | Significant between group difference favouring I group for exercise frequency (2.7 vs 3.1 sessions/week, p=0.012) at 2-mon. No other significant differences at 2 or 6-mon. |
| Johnson, 2017, TARGIT, [94] | USA | 17- ≤35yrs; 49% female; predominantly white | 330 | I: Behavioral weight management program via interactive technology + smoking cessation program. C: Smoking cessation program only. | Obesity, nutrition and physical activity + smoking | F2F, online, and technology (ipod, podcast, email and SMS) | 96 | Community | 24-mon: 68% | 0, 6, 12 and 24-m | Weight (kg) (measured) | No significant between group difference on weight. |
| Kim, 2018, [95] | USA | 17- ≤25yrs; 62% female; predominantly white | 187 | I: Provided wearable activity tracker with smartphone app for tracking, goal setting and feedback + participated in Physical Activity Instruction Program (PAIP) university course. C: PAIP only | Physical activity | I: F2F + wearable device. C: F2F | 15 | University | 15-wks: 44% | 0, 15 weeks | MVPA (accelerometer) | No significant between grp differences in MVPA. |
| Simons, 2018, [96] | Belgium | 17- ≤30yrs; 51% female; NR | 130 | I: Active Coach smartphone app (goals, tips, info, feedback) + wearable activity tracker. C: Received print-based generic physical activity information. | Physical activity | App + wearable device | 9 | Community | 21-wks: 84% | 0, 9 and 21 weeks | Objective & self-report PA (accelerometer/ self-report questionnaire) | No significant between group differences for any measure at 9 or 21 weeks (all p>.05). |
| Chiang, 2019, [97] | Taiwan | 17- ≤25yrs; NR; NR | 32 | I1: walking step goal of 12,000 steps per day. I2: walking step goal of 12,000 steps per day including 3 days per week on which walking at a step rate >103 steps/min. C: No intervention/ usual lifestyle | Physical activity | Wearable device + print materials | 8 | University | 2-mon: NR | 0 and 2-mon | Weight, BMI, hip circumference, visceral fat area, skeletal muscle mass (kg), body fat (%), waist circumference (measured – bioelectrical impedance analysis/tape measure) | Significant between group difference favouring I2 compared with I1 and C for visceral fat area (-13.1%, -0.18% and -0.97% respectively, p<0.05). No other significant between group differences |
| Halperin, 2019, [98] | Puerto Rico | 17- ≤25yrs; 72% female; NR | 40 | I: 10 weekly peer support groups focused on promoting dietary and physical activity changes, and stress reduction. C: usual care/ provision of basic educational resources on diet and physical activity | Nutrition and Physical activity + stress | F2F + smartphone app | 10 | University | 10-wks: 98%; 6-mon: 98% | 0, 2.5 and 6-mon | BMI (calculated from measured height and weight) | Significant between group difference in BMI favouring intervention at 10-wks (I: -0.8, C: +0.7 points, p<0.001) and maintained at 6-mon (I: -1.2, C: +0.8 points) |
| LaRose, 2019, [99] | USA | 17- ≤25yrs; 78% female; Predominantly white | 52 | Behavioural weight loss focusing on goal setting and changing high risk behaviours - 3 groups - I1: F2F. I2: web-based. I3: hybrid (F2F and web-based) | Obesity, Nutrition and Physical Activity + sleep and stress | I1: F2F  I2: Online  I3: F2F + Online | 12 | University | 3-mon: 81%; 6-mon: 75% | 0, 3 and 6-mon | Weight (% change) (measured) | No significant between group differences at either time point |
| Lyzwinski, 2019, [100] | Australia | 17- ≤25yrs; 67% female; Predominantly white | 90 | I1: mindfulness app (focused on weight loss through mindful eating, stress reduction and physical activity, and including behavioural self-monitoring electronic diary). I2: behavioural self-monitoring electronic diary | Obesity, Nutrition and Physical Activity + stress | I1: Smartphone app  I2: Online | 11 | University | 11-wks: 80% | 0, 11-wks | Weight (kg) (measured) | No significant between group difference in weight. |
| Pope, 2019, [101] | USA | 17- ≤35yrs; 74% female; Predominantly white | 38 | I: Smartwatch to track PA and Facebook group including nutrition and PA advice. C: Facebook group only (separate group to intervention participants) | Nutrition and Physical Activity | Wearable device + eHealth | 12 | University | 12-wks: 92% | 0, 12-wks | MVPA, leisure PA and sedentary time (accelerometer), cardiorespiratory fitness (3-min step test), body fat % (bioelectrical impedance), weight (measured), calories, fruit, vegetables, whole-grain and sugar sweetened beverages (self-administered 24-hr recall) | Between group differences not assessed, and statistical significance of within group changes not assessed. |
| Carfora, 2019, [102] | Italy | NR; 76% female; NR | 180 | I1: informational message - daily messages about health and environmental impact of excessive red and processed meat consumption (RPMC). I2: Emotional message - daily messages about health and environmental impact of excessive RPMC but presented in a way to evoke anticipated regret. Cl: daily messages about health and environmental consequences of eating sugar (received same information on RPMC after follow up) | Nutrition | eHealth | 2 | University | 2-wks: NR; 10-wks: 92% | 0, 2 and 10-wks | Red and processed meat intake (self-reported - short diet question at all timepoints and 2-week food diary at post intervention only) | Significantly lower intake in I1 and I2 compared with C at post intervention assessed via short diet question (7 vs 8 serves/ week) and food diary (6.50 vs 8.5 serves/week). Significantly lower intake in I2 compared with I1 and C at follow up assessed via short diet question (6 vs 7 serves/week). |
| Husband, 2019, [103] | Canada | 17- ≤25yrs; 72% female; NR | 20 | I1: indirect intervention group received information on the benefits of PA and behaviour change techniques e.g. planning. I2: direct intervention group received the same information plus information focusing on building self-identity around PA | Physical Activity | F2F | 6 | University | 6-wks: 92% | 0 and 6-wks | Leisure score index (calculated from moderate and vigorous PA) (self-report questionnaire) | Significant between group difference in leisure score index favouring I2 (change score - I1: 8.11 [95% CI −7.39–23.61], I2: 12.61 [95% CI −1.39–26.61]) |
| Maselli, 2019, [104] | Italy | 17- ≤30yrs; 61% female; NR | 33 | I1: Individual video counselling sessions (x7) informing of the importance of PA and providing knowledge and skills to increase PA, tailored to stage of change. One initial group session, and sent twice weekly emails with same information. I2: Wearable PA monitors which set a tailored PA goal and provide feedback, linked to webpage to plan/track PA. C: No intervention. | Physical Activity | I1: F2F + eHealth  I2: Wearable device + eHealth | 12 | University | 12-wks: 100%; 6-mon: 97% | 0 and 12-wks, and 6-mon | MET min/week (calculated from self-report questionnaire), MVPA min/week (accelerometer) | Significant increase in self-reported PA in I1 compared with I2 and C post intervention only (I1 +2063.3 MET min/week, I2 -309.6, C +751.91, p<0.001). No significant differences using accelerometer data at either timepoint. |
| Pfeffer, 2019, [105] | Germany | 17- ≤35yrs; 67% female; NR | 116 | I: 15 min one-on-one session to develop action and coping plans for physical activity for the next 7 days. C: Read a text from a popular scientific journal not related to physical activity | Physical Activity | F2F + Print materials | Single session | University | 1-week: 96% | 0 and 1-wk | MVPA hours/week (self-report questionnaire) | Significant between group difference favouring intervention (I: +1.63hrs/ week, C: +0.77hrs/ week, p=0.02). |
| Whatnall, 2019, [106] | Australia | 17- ≤35yrs; 67% female; NR | 124 | I: Brief nutrition intervention involving feedback, advice, goal setting and planning strategies to improve 4 target eating behaviours (fruit, veg, discretionary foods and breakfast). C: Brief alcohol intervention involving feedback and extra resources, and provided access to intervention after follow-up. | Nutrition | eHealth | Single session | University | 3-mon: 73% | 0 and 3-mon | Diet quality (self-report FFQ) | No significant between group difference in diet quality at 3-mon. |

**
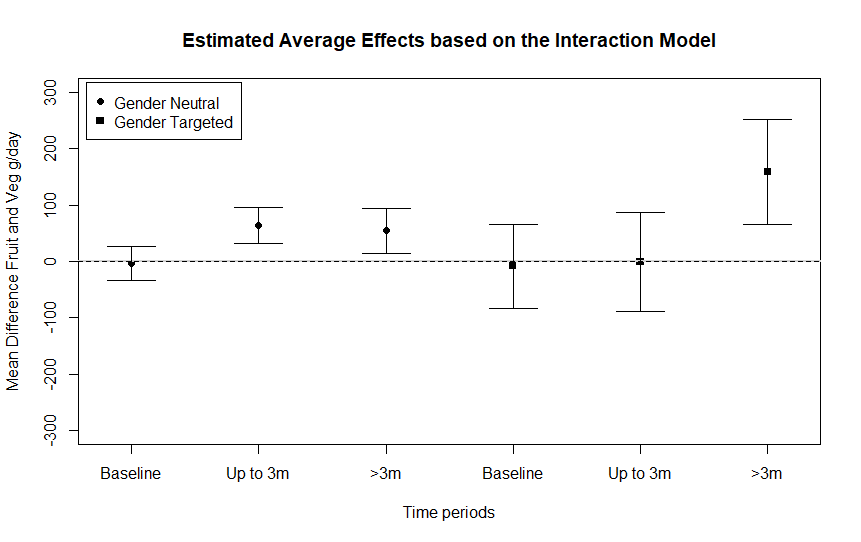
**

**Figure S1:** Mean differences by gender-neutral or gender targeted interventions and control arms in fruit and vegetable intake (g/day) over time.


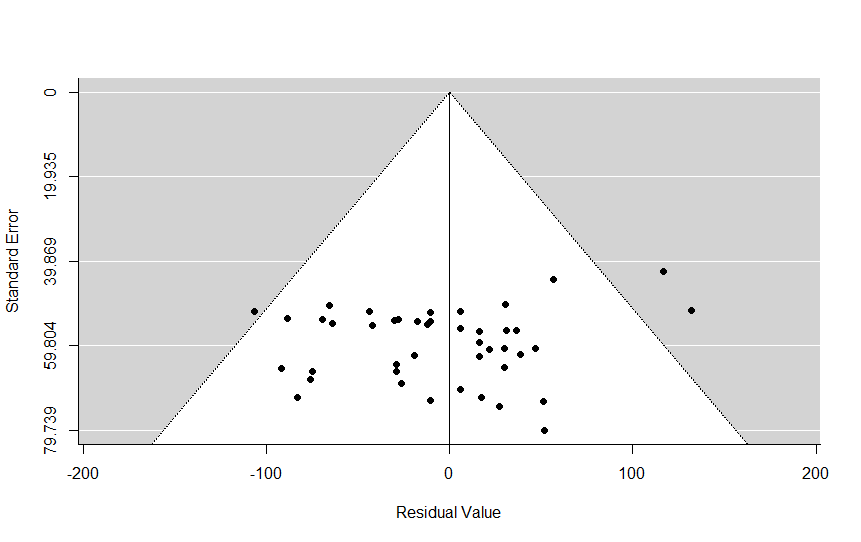


**Figure S2:** Funnel plot vs Standard Error – Fruit and Vegetables (g/day)

**
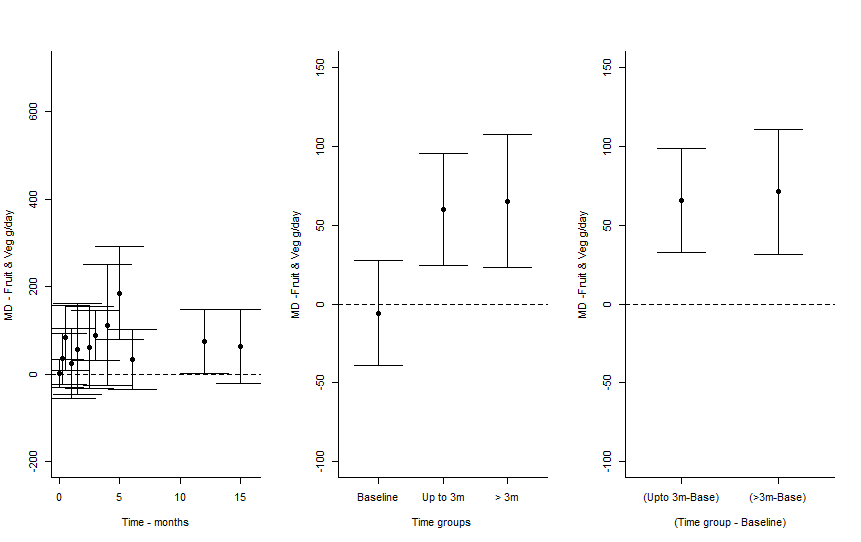
**

**Figure S3:** Plots of the means for effect – Fruit and Vegetables (g/day)


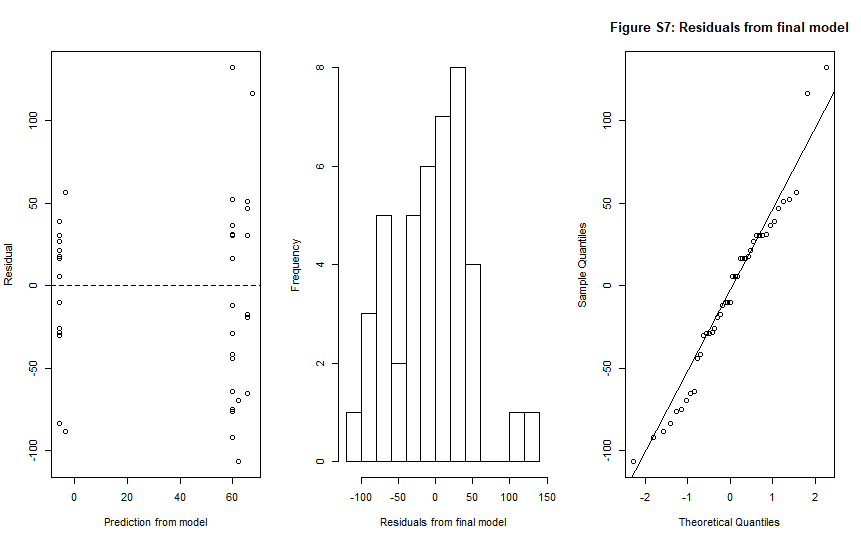


**Figure S4:** Model diagnostics – Fruit and Vegetables (g/day)


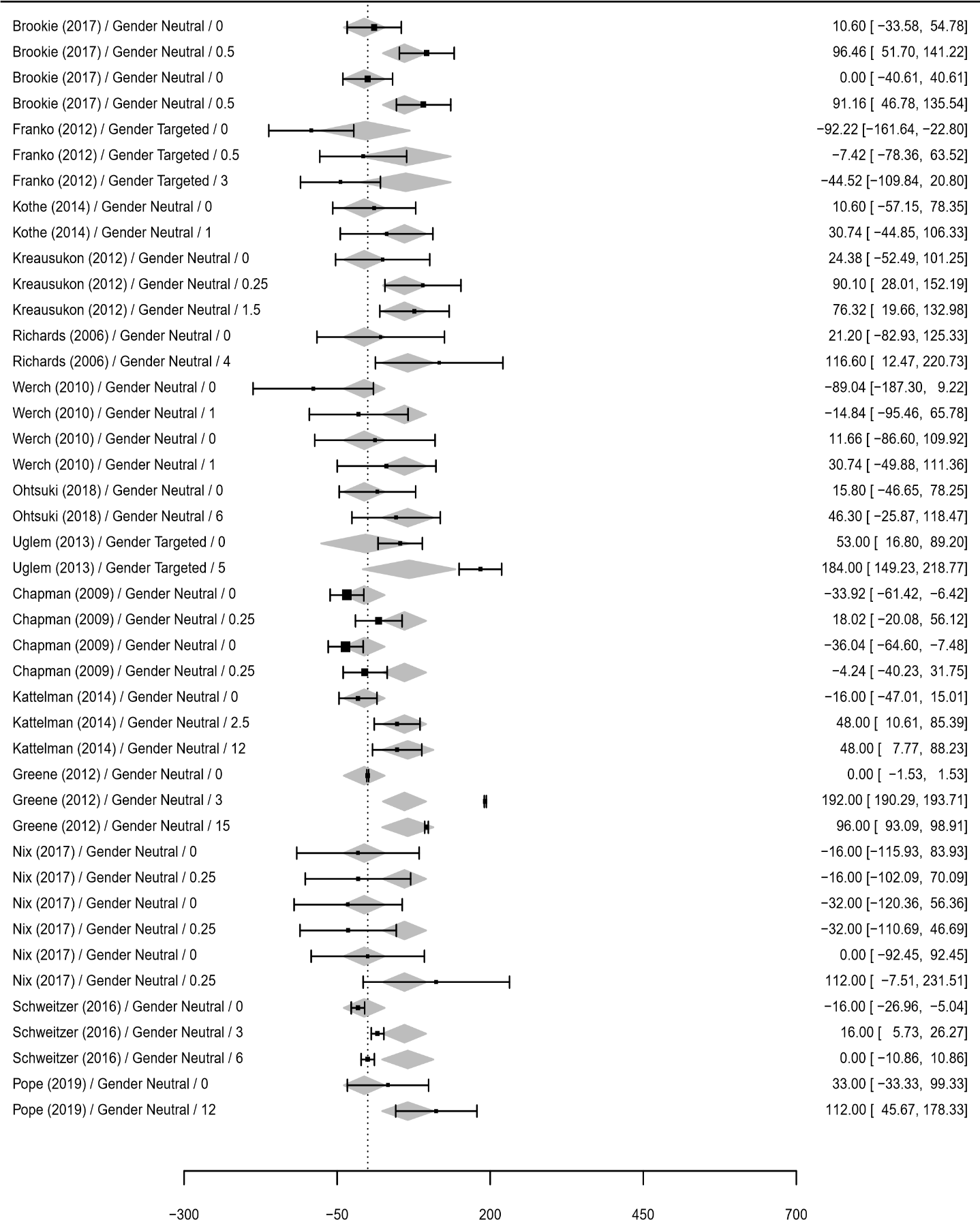


Mean Difference

**Figure S5:** Forest plot – fruit and vegetable intake (g/day)

**
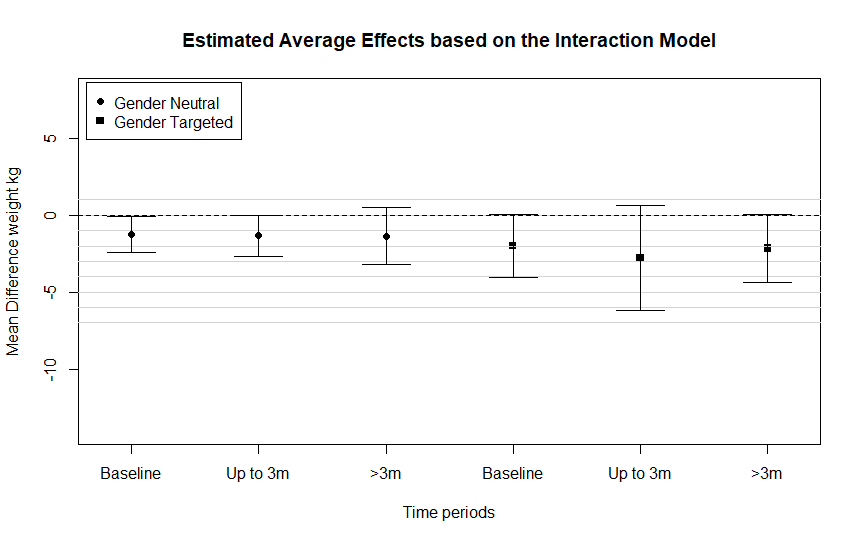
**

**Figure S6:** Mean differences by gender-neutral or gender targeted interventions and control arms in weight (kg) over time among weight gain prevention interventions.


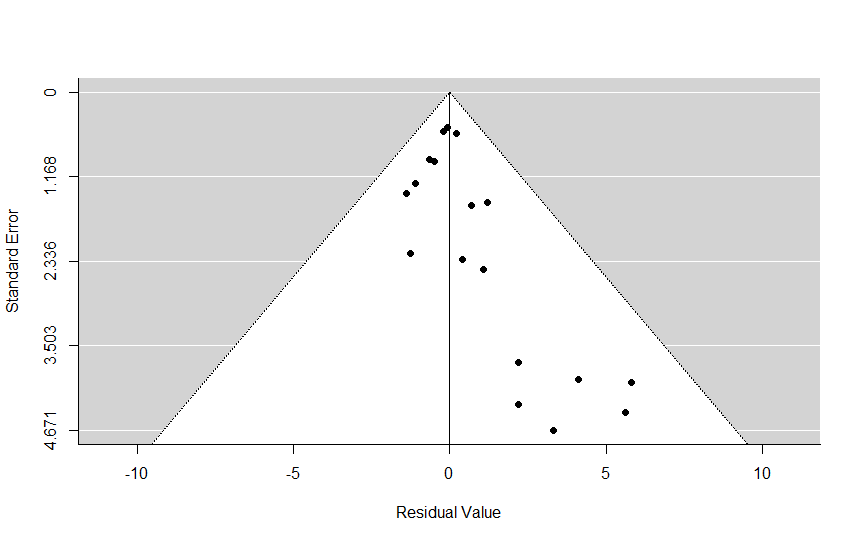
 **Figure S7:** Funnel plot vs Standard Error – Weight (kg) in weight gain prevention interventions.

**
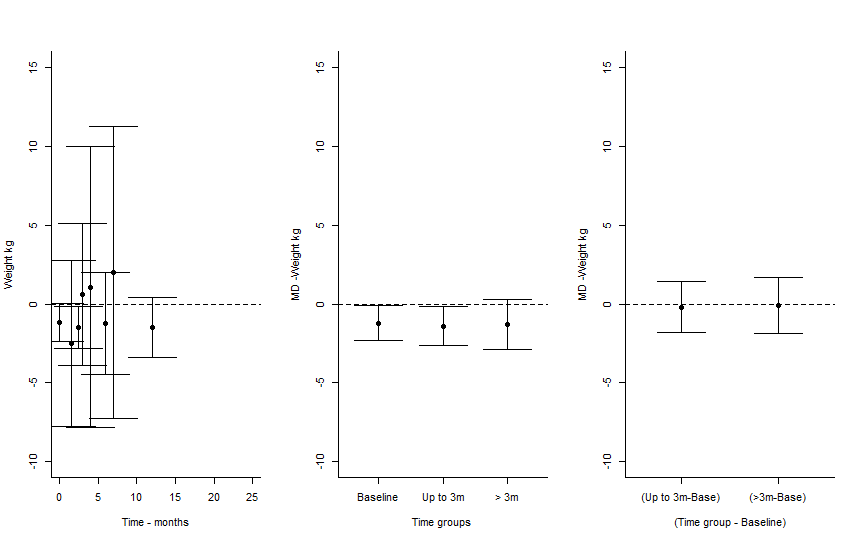
**

**Figure S8:** Plots of the means for effect – Weight (kg) in weight gain prevention interventions.

**
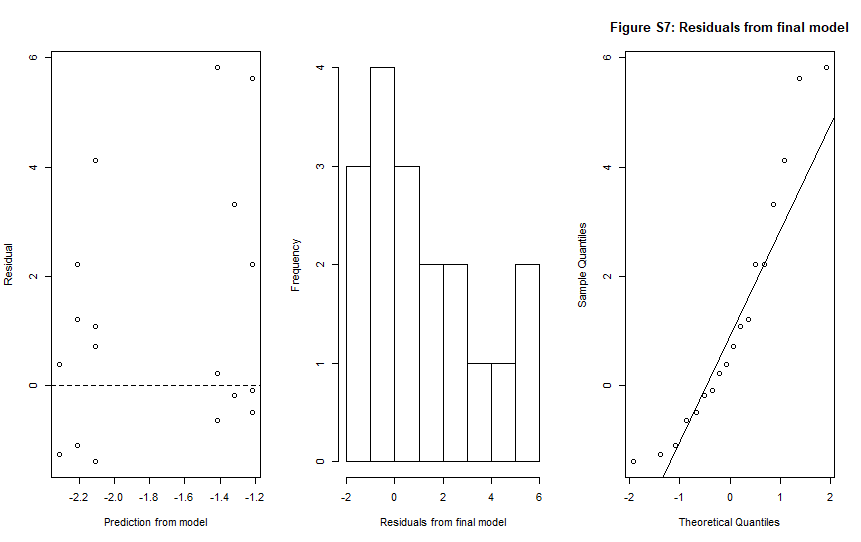
**

**Figure S9:** Model diagnostics – Weight (kg) in weight gain prevention interventions.


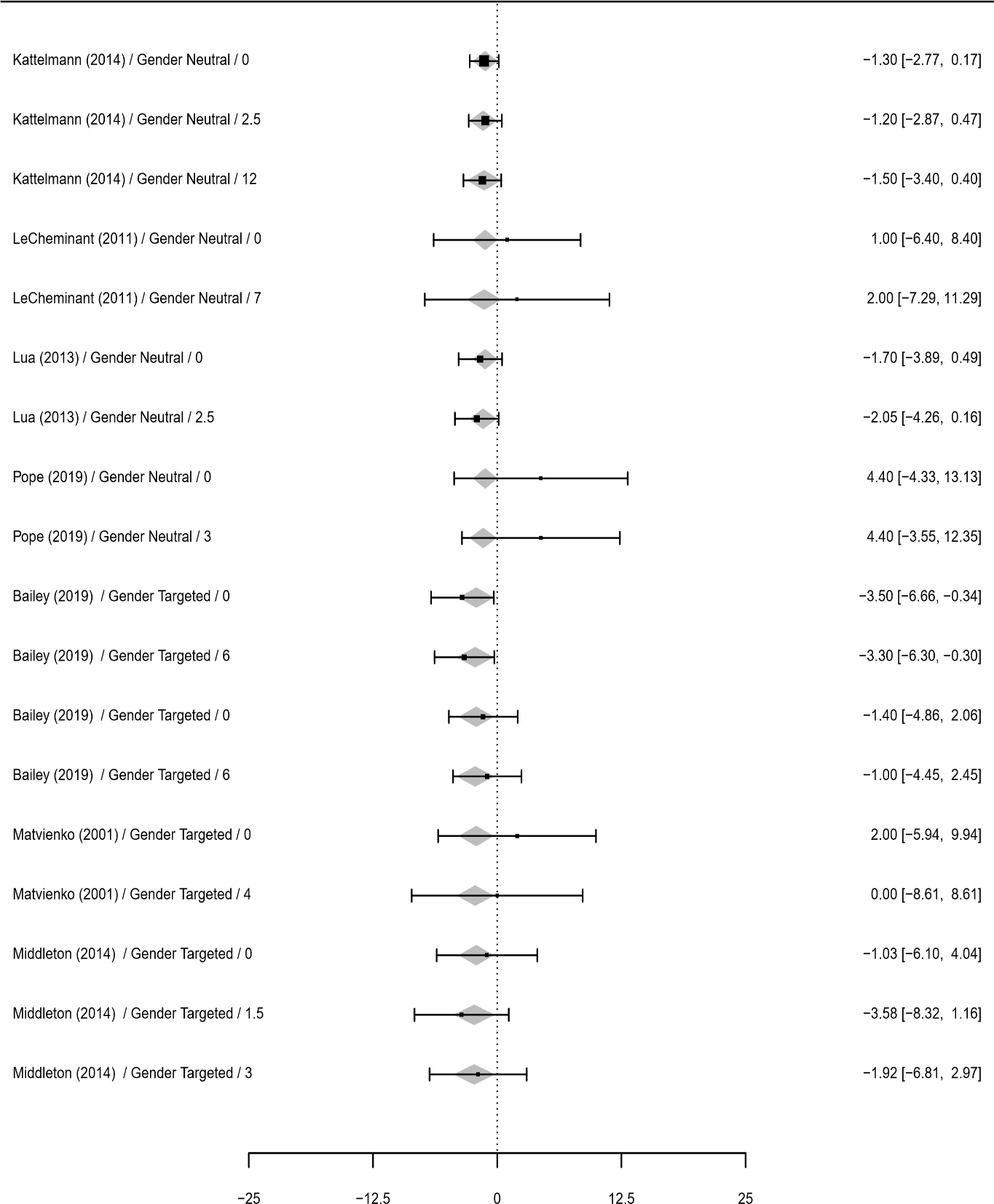


Mean Difference

**Figure S10:** Forest plot – weight (kg) in weight gain prevention interventions.

**
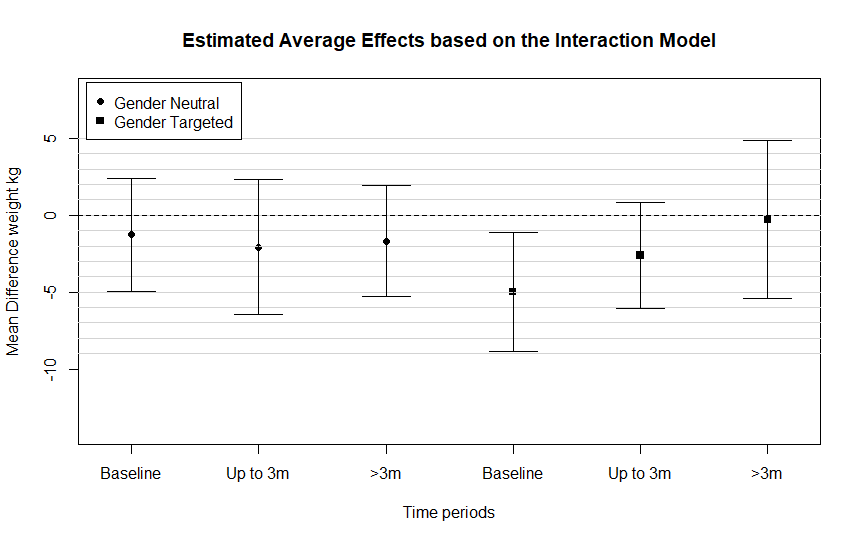
**

**Figure S11:** Mean differences by gender-neutral or gender targeted interventions and control arms in weight (kg) over time among weight loss studies

**
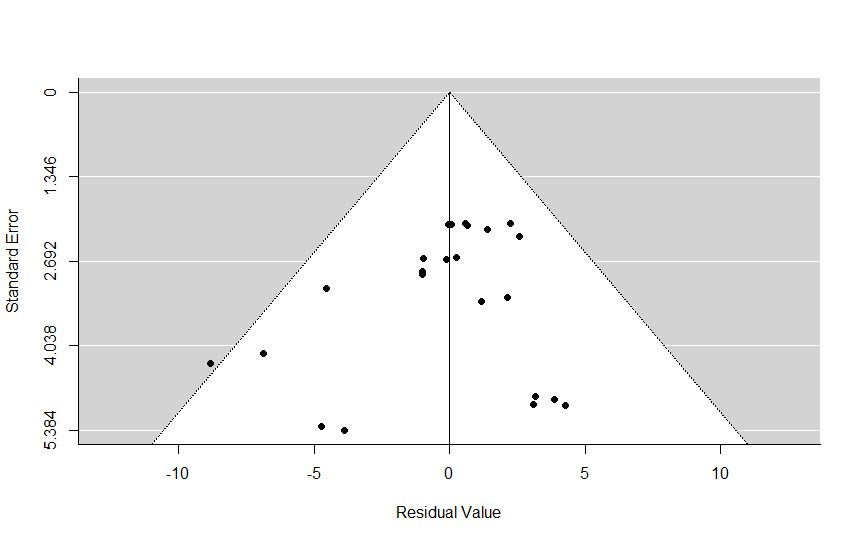
**

**Figure S12:** Funnel plot vs Standard Error – Weight (kg) in weight loss studies

**
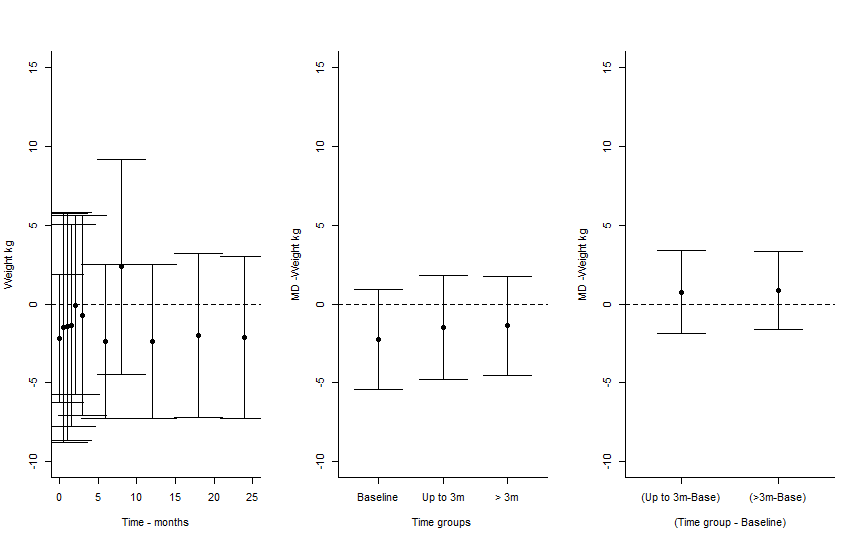
**

**Figure S13:** Plots of the means for effect – Weight (kg) in weight loss studies


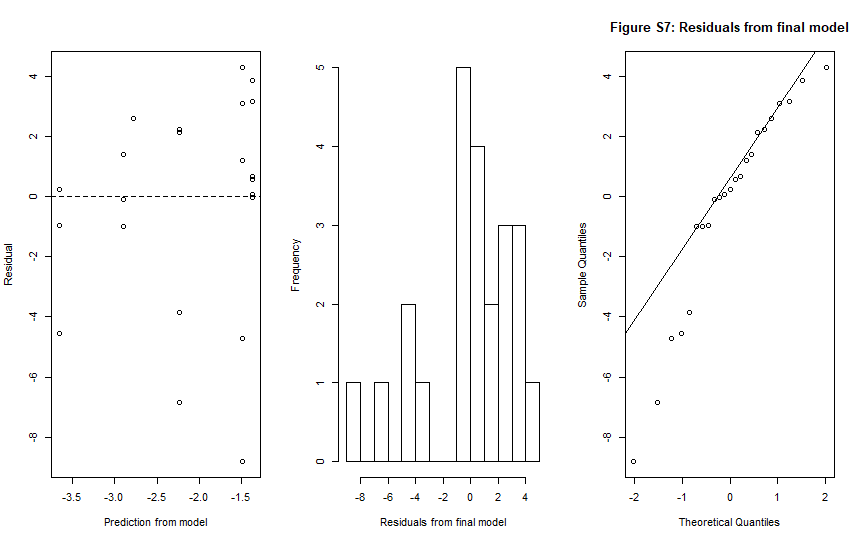


**Figure S14:** Model diagnostics – Weight (kg) in weight loss studies


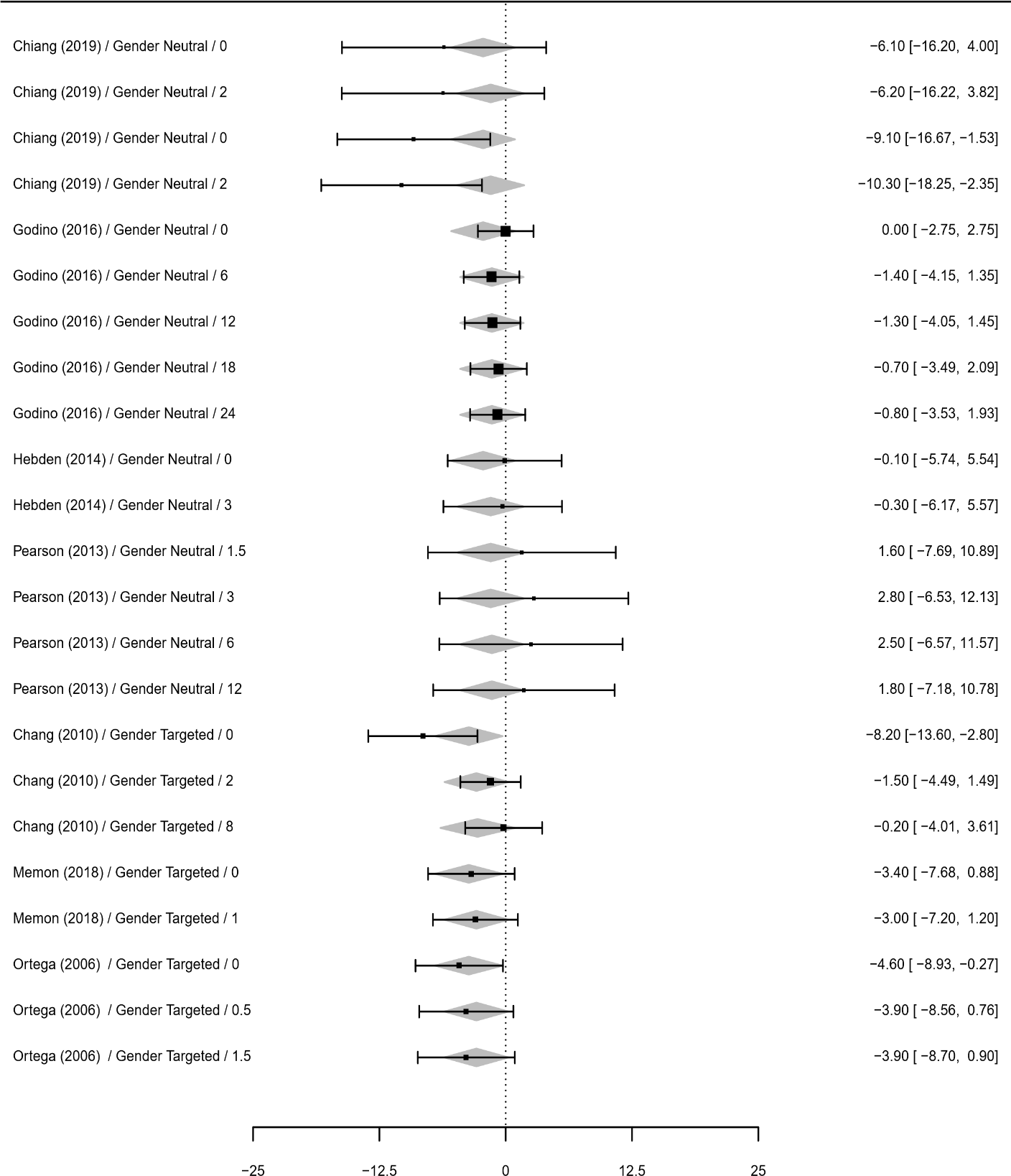


Mean Difference

**Figure S15:** Forest plot – weight (kg) in weight loss studies

**References**

1. Ashton LM, Morgan PJ, Hutchesson MJ, Rollo ME, Collins CE: **Feasibility and preliminary efficacy of the 'HEYMAN' healthy lifestyle program for young men: a pilot randomised controlled trial.** *Nutrition Journal* 2017, **16:**1-17.

2. Cambien F, Richard JL, Ducimetiere P, Warnet JM, Kahn J: **The Paris Cardiovascular Risk Factor Prevention Trial. Effects of two years of intervention in a population of young men.** *Journal of Epidemiology & Community Health* 1981, **35:**91-97.

3. Middleton KR, Perri MG: **A Randomized Trial Investigating the Effect of a Brief Lifestyle Intervention on Freshman-Year Weight Gain.** *Journal of American College Health* 2014, **62:**101-109.

4. Pellitteri K, Huberty J, Ehlers D, Bruening M: **Fit Minded College Edition Pilot Study: Can a Magazine-Based Discussion Group Improve Physical Activity in Female College Freshmen?** *Journal of Public Health Management & Practice* 2017, **23:**e10-e19.

5. Jung ME, Martin Ginis KA, Phillips SM, Lordon CD: **Increasing calcium intake in young women through gain-framed, targeted messages: A randomised controlled trial.** *Psychology & Health* 2011, **26:**531-547.

6. Franko DL, Jenkins A, Rodgers RF: **Toward Reducing Risk for Eating Disorders and Obesity in Latina College Women.** *Journal of Counseling and Development* 2012, **90:**298-307.

7. Share BL, Naughton GA, Obert P, Peat JK, Aumand EA, Kemp JG: **Effects of a Multi-Disciplinary Lifestyle Intervention on Cardiometabolic Risk Factors in Young Women with Abdominal Obesity: A Randomised Controlled Trial.** *PLoS ONE [Electronic Resource]* 2015, **10:**e0130270.

8. Stice E, Rohde P, Shaw H, Marti C: **Efficacy trial of a selective prevention program targeting both eating disorders and obesity among female college students: 1- and 2-year follow-up effects.** *Journal of Consulting and Clinical Psychology* 2013, **81:**183-189.

9. Tavakoli HR, Dini-Talatappeh H, Rahmati-Najarkolaei F, Fesharaki MG: **Efficacy of HBM-Based Dietary Education Intervention on Knowledge, Attitude, and Behavior in Medical Students.** *Iranian Red Crescent Medical Journal* 2016, **18**.

10. Chang MW, Nitzke S, Brown R: **Design and outcomes of a Mothers In Motion behavioral intervention pilot study.** *Journal of Nutrition Education & Behavior* 2010, **42:**S11-21.

11. Eiben G, Lissner L: **Health Hunters--an intervention to prevent overweight and obesity in young high-risk women.** 2006, **30:**691-696.

12. Ortega RM, Rodriguez-Rodriguez E, Aparicio A, Marin-Arias LI, Lopez-Sobaler AM: **Responses to two weight-loss programs based on approximating the diet to the ideal: Differences associated with increased cereal or vegetable consumption.** *International Journal for Vitamin and Nutrition Research* 2006, **76:**367-376.

13. Williams DR, Lewis NM: **Effectiveness of nutrition counseling in young adult males.** *Nutrition Research* 2002, **22:**911-917.

14. Uglem S, Stea TH, Kjollesdal MKR, Frolich W, Wandel M: **A nutrition intervention with a main focus on vegetables and bread consumption among young men in the Norwegian National Guard.** *Food & Nutrition Research* 2013, **57**.

15. Hutchesson M, Callister R, Morgan P, Pranata I, Clarke E, Skinner G, Ashton L, Whatnall M, Jones M, Oldmeadow C, Collins C: **A Targeted and Tailored eHealth Weight Loss Program for Young Women: The Be Positive Be Healthe Randomized Controlled Trial.** *Healthcare* 2018, **6:**39.

16. Jauho AM, Pyky R, Ahola R, Kangas M, Virtanen P, Korpelainen R, Jamsa T: **Effect of wrist-worn activity monitor feedback on physical activity behavior: A randomized controlled trial in Finnish young men.** *Preventive Medicine Reports* 2015, **2:**628-634.

17. Katterman SN, Butryn ML, Hood MM, Lowe MR: **Daily weight monitoring as a method of weight gain prevention in healthy weight and overweight young adult women.** *Journal of Health Psychology* 2016, **21:**2955-2965.

18. Katterman SN, Goldstein SP, Butryn ML, Forman EM, Lowe MR: **Efficacy of an acceptance-based behavioral intervention for weight gain prevention in young adult women.** *Journal of Contextual Behavioral Science* 2014, **3:**45-50.

19. Klem ML, Viteri JE, Wing RR: **Primary prevention of weight gain for women aged 25-34: the acceptability of treatment formats.** *International Journal of Obesity* 2000, **24:**219-225.

20. Ornes L, Ransdell LB: **Web-based physical activity intervention for college-aged women.** *International Electronic Journal of Health Education* 2007, **10:**126-137.

21. Rote AE, Klos LA, Brondino MJ, Harley AE, Swartz AM: **The Efficacy of a Walking Intervention Using Social Media to Increase Physical Activity: A Randomized Trial.** *Journal of Physical Activity & Health* 2015, **12:**S18-S25.

22. Sriramatr S, Berry TR, Spence JC: **An Internet-based intervention for promoting and maintaining physical activity: a randomized controlled trial.** *American Journal of Health Behavior* 2014, **38:**430-439.

23. Tobias LL, MacDonald ML: **Internal locus of control and weight loss: An insufficient condition.** *Journal of Consulting and Clinical Psychology* 1977, **45:**647-653.

24. Butryn ML, Forman E, Hoffman K, Shaw J, Juarascio A: **A pilot study of acceptance and commitment therapy for promotion of physical activity.** *Journal of Physical Activity & Health* 2011, **8:**516-522.

25. Valve P, Lehtinen-Jacks S, Eriksson T, Lehtinen M, Lindfors P, Saha MT, Rimpela A, Angle S: **LINDA - a solution-focused low-intensity intervention aimed at improving health behaviors of young females: a cluster-randomized controlled trial.** *BMC Public Health* 2013, **13:**1044.

26. Leinonen AM, Pyky R, Ahola R, Kangas M, Siirtola P, Luoto T, Enwald H, Ikaheimo TM, Roning J, Keinanen-Kiukaanniemi S, et al: **Feasibility of Gamified Mobile Service Aimed at Physical Activation in Young Men: Population-Based Randomized Controlled Study (MOPO).** *JMIR Mhealth Uhealth* 2017, **5:**e146.

27. Matvienko O, Lewis DS, Schafer E: **A college nutrition science course as an intervention to prevent weight gain in female college freshmen.** *Journal of Nutrition Education* 2001, **33:**95-101.

28. Bailey BW, Bartholomew CL, Summerhays C, Deru L, Compton S, Tucker LA, LeCheminant JD, Hicks J: **The Impact of Step Recommendations on Body Composition and Physical Activity Patterns in College Freshman Women: A Randomized Trial.** *J Obes* 2019, **2019:**4036825.

29. Memon AR, Masood T, Awan WA, Waqas A: **The effectiveness of an incentivized physical activity programme (Active Student) among female medical students in Pakistan: A Randomized Controlled Trial.** *J Pak Med Assoc* 2018, **68:**1438-1445.

30. Amiot CE, El Hajj Boutros G, Sukhanova K, Karelis AD: **Testing a novel multicomponent intervention to reduce meat consumption in young men.** *PloS one* 2018, **13:**e0204590-e0204590.

31. Bertz F, Pacanowski CR, Levitsky DA: **Frequent Self-Weighing with Electronic Graphic Feedback to Prevent Age-Related Weight Gain in Young Adults.** *Obesity* 2015, **23:**2009-2014.

32. Brookie KL, Mainvil LA, Carr AC, Vissers MC, Conner TS: **The development and effectiveness of an ecological momentary intervention to increase daily fruit and vegetable consumption in low-consuming young adults.** *Appetite* 2017, **108:**32-41.

33. Brown ON, O'Connor LE, Savaiano D: **Mobile MyPlate: a pilot study using text messaging to provide nutrition education and promote better dietary choices in college students.** *Journal of American College Health* 2014, **62:**320-327.

34. Buscemi J, Yurasek AM, Dennhardt AA, Martens MP, Murphy JG: **A randomized trial of a brief intervention for obesity in college students.** *Clinical Obesity* 2011, **1:**131-140.

35. Chapman J, Armitage CJ, Norman P: **Comparing implementation intention interventions in relation to young adults' intake of fruit and vegetables.** *Psychology & Health* 2009, **24:**317-332.

36. Hivert M, Langlois M, Berard P, Cuerrier J, Carpentier A: **Prevention of weight gain in young adults through a seminar-based intervention program.** *International Journal of Obesity* 2007, **31:**1262-1269.

37. Werch CE, Moore MJ, Bian H, DiClemente CC, Huang IC, Ames SC, Thombs D, Weiler RM, Pokorny SB: **Are effects from a brief multiple behavior intervention for college students sustained over time?** *Preventive Medicine* 2010, **50:**30-34.

38. Zhang Y, Cooke R: **Using a combined motivational and volitional intervention to promote exercise and healthy dietary behaviour among undergraduates.** *Diabetes Research & Clinical Practice* 2012, **95:**215-223.

39. LaChausse RG: **My student body: Effects of an internet-based prevention program to decrease obesity among college students.** *Journal of American College Health* 2012, **60:**324-330.

40. LaRose JG, Tate DF, Gorin AA, Wing RR: **Preventing weight gain in young adults: A randomized controlled pilot study.** *American Journal of Preventive Medicine* 2010, **39:**63-68.

41. Laska MN, Lytle LA, Nanney MS, Moe SG, Linde JA, Hannan PJ: **Results of a 2-year randomized, controlled obesity prevention trial: Effects on diet, activity and sleep behaviors in an at-risk young adult population.** *Preventive Medicine: An International Journal Devoted to Practice and Theory* 2016, **89:**230-236.

42. LeCheminant JD, Smith JD, Covington NK, Hardin-Renschen T, Heden T: **Pedometer use in university freshmen: a randomized controlled pilot study.** *American Journal of Health Behavior* 2011, **35:**777-784.

43. Lhakhang P, Godinho C, Knoll N, Schwarzer R: **A brief intervention increases fruit and vegetable intake. A comparison of two intervention sequences.** *Appetite* 2014, **82:**103-110.

44. Lua PL, Wan Dali WPE, Shahril MR: **Multimodal Nutrition Education Intervention: A Cluster Randomised Controlled Trial Study on Weight Gain and Physical Activity Pattern Among University Students in Terengganu, Malaysia.** *Malaysian Journal of Nutrition* 2013, **19:**339-352.

45. Maher JP, Conroy DE: **Habit strength moderates the effects of daily action planning prompts on physical activity but not sedentary behavior.** *Journal of Sport & Exercise Psychology* 2015, **37:**97-107.

46. Martens MP, Buscemi J, Smith AE, Murphy JG: **The short-term efficacy of a brief motivational intervention designed to increase physical activity among college students.** *Journal of Physical Activity & Health* 2012, **9:**525-532.

47. Meng J, Peng W, Shin SY, Chung M: **Online Self-Tracking Groups to Increase Fruit and Vegetable Intake: A Small-Scale Study on Mechanisms of Group Effect on Behavior Change.** *Journal of Medical Internet Research* 2017, **19:**e63.

48. Napolitano MA, Hayes S, Bennett GG, Ives AK, Foster GD: **Using Facebook and text messaging to deliver a weight loss program to college students.** *Obesity* 2013, **21:**25-31.

49. Nix E, Wengreen HJ: **Social approval bias in self-reported fruit and vegetable intake after presentation of a normative message in college students.** *Appetite* 2017, **116:**552-558.

50. O'Brien LM, Palfai TP: **Efficacy of a brief web-based intervention with and without SMS to enhance healthy eating behaviors among university students.** *Eating Behaviors* 2016, **23:**104-109.

51. Park A, Nitzke S, Kritsch K, Kattelmann K, White A, Boeckner L, Lohse B, Hoerr S, Greene G, Zhang Z: **Internet-based interventions have potential to affect short-term mediators and indicators of dietary behavior of young adults.** *Journal of Nutrition Education and Behavior* 2008, **40:**288-297.

52. Pearson ES, Irwin JD, Morrow D, Battram DS, Melling CW: **The CHANGE program: comparing an interactive vs. prescriptive approach to self-management among university students with obesity.** *Canadian Journal of Diabetes* 2013, **37:**4-11.

53. Kypri K, McAnally HM: **Randomized controlled trial of a web-based primary care intervention for multiple health risk behaviors.** *Preventive Medicine* 2005, **41:**761-766.

54. Gokee-LaRose J, Gorin AA, Wing RR: **Behavioral self-regulation for weight loss in young adults: A randomized controlled trial.** *The International Journal of Behavioral Nutrition and Physical Activity Vol 6 2009, ArtID 10* 2009, **6**.

55. Gow RW, Trace SE, Mazzeo SE: **Preventing weight gain in first year college students: An online intervention to prevent the "freshman fifteen.".** *Eating Behaviors* 2010, **11:**33-39.

56. Hebden L, Chey T, Allman-Farinelli M: **Lifestyle intervention for preventing weight gain in young adults: a systematic review and meta-analysis of RCTs.** *Obes Rev* 2012, **13:**692-710.

57. Kerr DA, Harray AJ, Pollard CM, Dhaliwal SS, Delp EJ, Howat PA, Pickering MR, Ahmad Z, Meng X, Pratt IS, et al: **The connecting health and technology study: A 6-month randomized controlled trial to improve nutrition behaviours using a mobile food record and text messaging support in young adults.** *The International Journal of Behavioral Nutrition and Physical Activity Vol 13 2016, ArtID 52* 2016, **13**.

58. Allman-Farinelli M, Partridge SR, McGeechan K, Balestracci K, Hebden L, Wong A, Phongsavan P, Denney-Wilson E, Harris MF, Bauman A: **A Mobile Health Lifestyle Program for Prevention of Weight Gain in Young Adults (TXT2BFiT): Nine-Month Outcomes of a Randomized Controlled Trial.** *Jmir Mhealth and Uhealth* 2016, **4:**408-419.

59. Svetkey LP, Batch BC, Lin PH, Intille SS, Corsino L, Tyson CC, Bosworth HB, Grambow SC, Voils C, Loria C, et al: **Cell phone intervention for you (CITY): A randomized, controlled trial of behavioral weight loss intervention for young adults using mobile technology.[Erratum appears in Obesity (Silver Spring). 2016 Feb;24(2):536 Note: Bennett, G B [Corrected to Bennett, G G]; PMID: 26813533].** *Obesity* 2015, **23:**2133-2141.

60. Kattelmann KK, Bredbenner CB, White AA, Greene GW, Hoerr SL, Kidd T, Colby S, Horacek TM, Phillips BW, Koenings MM, et al: **The effects of Young Adults Eating and Active for Health (YEAH): A theory-based web-delivered intervention.** *Journal of Nutrition Education and Behavior* 2014, **46:**S27-S41.

61. Do M, Kattelmann K, Boeckner L, Greene G, White A, Hoerr S, Horacek T, Lohse B, Phillips B, Nitzke S: **Low-income young adults report increased variety in fruit and vegetable intake after a stage-tailored intervention.** *Nutrition Research* 2008, **28:**517-522.

62. Franko DL, Cousineau TM, Trant M, Green TC, Rancourt D, Thompson D, Ainscough J, Mintz LB, Ciccazzo M: **Motivation, self-efficacy, physical activity and nutrition in college students: Randomized controlled trial of an internet-based education program.** *Preventive Medicine: An International Journal Devoted to Practice and Theory* 2008, **47:**369-377.

63. Goodman S, Morrongiello B, Meckling K: **A randomized, controlled trial evaluating the efficacy of an online intervention targeting vitamin D intake, knowledge and status among young adults.** *International Journal of Behavioral Nutrition & Physical Activity* 2016, **13:**116.

64. Heeren GA, Jemmott JB, Marange CS, Rumosa Gwaze A, Batidzirai JM, Ngwane Z, Mandeya A, Tyler JC: **Health-Promotion Intervention Increases Self-Reported Physical Activity in Sub-Saharan African University Students: A Randomized Controlled Pilot Study.** *Behavioral Medicine* 2017**:**1-9.

65. Kendzierski D, Ritter RL, Stump TK, Anglin CL: **The effectiveness of an implementation intentions intervention for fruit and vegetable consumption as moderated by self-schema status.** *Appetite* 2015, **95:**228-238.

66. Knauper B, McCollam A, Rosen-Brown A, Lacaille J, Kelso E, Roseman M: **Fruitful plans: Adding targeted mental imagery to implementation intentions increases fruit consumption.** *Psychology & Health* 2011, **26:**601-617.

67. Kothe EJ, Mullan BA: **A randomised controlled trial of a theory of planned behaviour to increase fruit and vegetable consumption. Fresh Facts.** *Appetite* 2014, **78:**68-75.

68. Kreausukon P, Gellert P, Lippke S, Schwarzer R: **Planning and self-efficacy can increase fruit and vegetable consumption: a randomized controlled trial.** *Journal of Behavioral Medicine* 2012, **35:**443-451.

69. Richards A, Kattelmann KK, Ren C: **Motivating 18- to 24-year-olds to increase their fruit and vegetable consumption.** *Journal of the American Dietetic Association* 2006, **106:**1405-1411.

70. Rompotis CJ, Grove JR, Byrne SM: **Benefits of habit-based informational interventions: a randomised controlled trial of fruit and vegetable consumption.** *Australian & New Zealand Journal of Public Health* 2014, **38:**247-252.

71. Sandrick J, Tracy D, Eliasson A, Roth A, Bartel J, Simko M, Bowman T, Harouse-Bell K, Kashani M, Vernalis M: **Effect of a Counseling Session Bolstered by Text Messaging on Self-Selected Health Behaviors in College Students: A Preliminary Randomized Controlled Trial.** *Jmir Mhealth and Uhealth* 2017, **5**.

72. Schweitzer AL, Ross JT, Klein CJ, Lei KY, Mackey ER: **An Electronic Wellness Program to Improve Diet and Exercise in College Students: A Pilot Study.** *Jmir Research Protocols* 2016, **5**.

73. Stephens JD, Yager AM, Allen J: **Smartphone technology and text messaging for weight loss in young adults: A randomized controlled trial.** *Journal of Cardiovascular Nursing* 2017, **32:**39-46.

74. Annesi JJ, Howton A, Johnson PH, Porter KJ: **Pilot testing a cognitive-behavioral protocol on psychosocial predictors of exercise, nutrition, weight, and body satisfaction changes in a college-level health-related fitness course.** *Journal of American College Health* 2015, **63:**268-278.

75. Greene GW, White AA, Hoerr SL, Lohse B, Schembre SM, Riebe D, Patterson J, Kattelmann KK, Shoff S, Horacek T, et al: **Impact of an Online Healthful Eating and Physical Activity Program for College Students.** *American Journal of Health Promotion* 2012, **27:**E47-E58.

76. Ohtsuki M, Shibata K, Fukuwatari T, Sasaki Y, Nakai K: **Randomized controlled trial of educational intervention to increase consumption of vegetables by Japanese university students.** *Health Education* 2018, **118:**290-303.

77. Kothe EJ, Mullan BA, Butow P: **Promoting fruit and vegetable consumption. Testing an intervention based on the theory of planned behaviour.** *Appetite* 2012, **58:**997-1004.

78. Godino JG, Merchant G, Norman GJ, Donohue MC, Marshall SJ, Fowler JH, Calfas KJ, Huang JS, Rock CL, Griswold WG, et al: **Using social and mobile tools for weight loss in overweight and obese young adults (Project SMART): a 2 year, parallel-group, randomised, controlled trial.** *The Lancet Diabetes and Endocrinology* 2016, **4:**747-755.

79. Pope L, Harvey J: **The Efficacy of Incentives to Motivate Continued Fitness-Center Attendance in College First-Year Students: A Randomized Controlled Trial.** *Journal of American College Health* 2014, **62:**81-90.

80. Walsh JC, Corbett T, Hogan M, Duggan J, McNamara A: **An mHealth Intervention Using a Smartphone App to Increase Walking Behavior in Young Adults: A Pilot Study.** *Jmir Mhealth and Uhealth* 2016, **4**.

81. Conner M, Rhodes RE, Morris B, McEachan R, Lawton R: **Changing exercise through targeting affective or cognitive attitudes.** *Psychology & Health* 2011, **26:**133-149.

82. Cooke PA, Tully MA, Cupples ME, Gilliland AE, Gormley GJ: **A randomised control trial of experiential learning to promote physical activity.** *Education for Primary Care* 2013, **24:**427-435.

83. Cooke R, Trebaczyk H, Harris P, Wright AJ: **Self-affirmation promotes physical activity.** *Journal of Sport & Exercise Psychology* 2014, **36:**217-223.

84. Eisenberg MH, Phillips L, Fowler L, Moore PJ: **The impact of E-diaries and accelerometers on young adults' perceived and objectively assessed physical activity.** *Psychology of Sport and Exercise* 2017, **30:**55-63.

85. Jakicic JM, Davis KK, Rogers RJ, King WC, Marcus MD, Helsel D, Rickman AD, Wahed AS, Belle SH: **Effect of wearable technology combined with a lifestyle intervention on long-term weight loss: The IDEA randomized clinical trial.** *JAMA - Journal of the American Medical Association* 2016, **316:**1161-1171.

86. Phimarn W, Paktipat P, Pansiri K, Klabklang P, DuangJanchot P, Tongkul A: **Effect of Weight Control Counselling in Overweight and Obese Young Adults.** *Indian Journal of Pharmaceutical Sciences* 2017, **79:**35-41.

87. Sharp P, Caperchione C: **The effects of a pedometer-based intervention on first-year university students: A randomized control trial.** *Journal of American College Health* 2016, **64:**630-638.

88. Strohacker K, Galarraga O, Emerson J, Fricchione SR, Lohse M, Williams DM: **Impact of small monetary incentives on exercise in university students.** *American Journal of Health Behavior* 2015, **39:**779-786.

89. Bray SR, Beauchamp MR, Latimer AE, Hoar SD, Shields CA, Bruner MW: **Effects of a print-mediated intervention on physical activity during transition to the first year of university.** *Behavioral Medicine* 2011, **37:**60-69.

90. Calfas KJ, Sallis JF, Nichols JF, Sarkin JA, Johnson MF, Caparosa S, Thompson S, Gehrman CA, Alcaraz JE: **Project GRAD: two-year outcomes of a randomized controlled physical activity intervention among young adults. Graduate Ready for Activity Daily.** *American Journal of Preventive Medicine* 2000, **18:**28-37.

91. Wing RR, Tate DF, Espeland MA, Lewis CE, LaRose JG, Gorin AA, Bahnson J, Perdue LH, Hatley KE, Ferguson E, et al: **Innovative Self-Regulation Strategies to Reduce Weight Gain in Young Adults: The Study of Novel Approaches to Weight Gain Prevention (SNAP) Randomized Clinical Trial.** *JAMA Internal Medicine* 2016, **176:**755-762.

92. Weinstock J, Capizzi J, Weber SM, Pescatello LS, Petry NM: **Exercise as an intervention for sedentary hazardous drinking college students: A pilot study.** *Mental Health and Physical Activity* 2014, **7:**55-62.

93. Weinstock J, Petry NM, Pescatello LS, Henderson CE: **Sedentary college student drinkers can start exercising and reduce drinking after intervention.** *Psychology of Addictive Behaviors* 2016, **30:**791-801.

94. Johnson KC, Thomas F, Richey P, Tran QT, Tylavsky F, Miro D, Coday M: **The Primary Results of the Treating Adult Smokers at Risk for Weight Gain with Interactive Technology (TARGIT) Study.** *Obesity (Silver Spring)* 2017, **25:**1691-1698.

95. Kim Y, Lumpkin A, Lochbaum M, Stegemeier S, Kitten K: **Promoting physical activity using a wearable activity tracker in college students: A cluster randomized controlled trial.** *J Sports Sci* 2018, **36:**1889-1896.

96. Simons D, De Bourdeaudhuij I, Clarys P, De Cocker K, Vandelanotte C, Deforche B: **Effect and Process Evaluation of a Smartphone App to Promote an Active Lifestyle in Lower Educated Working Young Adults: Cluster Randomized Controlled Trial.** *JMIR Mhealth Uhealth* 2018, **6:**e10003.

97. Chiang T-L, Chen C, Hsu C-H, Lin Y-C, Wu H-J: **Is the goal of 12,000 steps per day sufficient for improving body composition and metabolic syndrome? The necessity of combining exercise intensity: a randomized controlled trial.** *BMC Public Health* 2019, **19:**1215.

98. Halperin DT, Laux J, LeFranc-García C, Araujo C, Palacios C: **Findings From a Randomized Trial of Weight Gain Prevention Among Overweight Puerto Rican Young Adults.** *J Nutr Educ Behav* 2019, **51:**205-216.

99. LaRose JG, Tate DF, Lanoye A, Fava JL, Jelalian E, Blumenthal M, Caccavale LJ, Wing RR: **Adapting evidence-based behavioral weight loss programs for emerging adults: A pilot randomized controlled trial.** *J Health Psychol* 2019, **24:**870-887.

100. Lyzwinski LN, Caffery L, Bambling M, Edirippulige S: **The Mindfulness App Trial for Weight, Weight-Related Behaviors, and Stress in University Students: Randomized Controlled Trial.** *JMIR Mhealth Uhealth* 2019, **7:**e12210.

101. Pope ZC, Barr-Anderson DJ, Lewis BA, Pereira MA, Gao Z: **Use of Wearable Technology and Social Media to Improve Physical Activity and Dietary Behaviors among College Students: A 12-Week Randomized Pilot Study.** *Int J Environ Res Public Health* 2019, **16**.

102. Carfora V, Bertolotti M, Catellani P: **Informational and emotional daily messages to reduce red and processed meat consumption.** *Appetite* 2019, **141:**104331.

103. Husband CJ, Wharf-Higgins J, Rhodes RE: **A feasibility randomized trial of an identity-based physical activity intervention among university students.** *Health Psychology and Behavioral Medicine* 2019, **7:**128-146.

104. Maselli M, Gobbi E, Carraro A: **Effectiveness of individual counseling and activity monitors to promote physical activity among university students.** *J Sports Med Phys Fitness* 2019, **59:**132-140.

105. Pfeffer I, Strobach T: **Effects of a planning intervention on physical activity behavior in an RCT: Intention strength as moderator and action planning, coping planning, and coping self-efficacy as mediators.** *Sport, Exercise, and Performance Psychology* 2019, **8:**192-209.

106. Whatnall MC, Patterson AJ, Chiu S, Oldmeadow C, Hutchesson MJ: **Feasibility and Preliminary Efficacy of the Eating Advice to Students (EATS) Brief Web-Based Nutrition Intervention for Young Adult University Students: A Pilot Randomized Controlled Trial.** *Nutrients* 2019, **11**.
